# A therapeutic antisense oligonucleotide encompassing 2′-*O*-methoxyethyl modification triggers unique perturbation of the transcriptome

**DOI:** 10.1093/narmme/ugag002

**Published:** 2026-01-06

**Authors:** Eric W Ottesen, Wren A Murzyn, Robert L Kaas, Keaton J Bertrand, Jessica L Payne, Ravindra N Singh

**Affiliations:** Department of Biomedical Sciences, Iowa State University, Ames, IA 50011,United States; Department of Biomedical Sciences, Iowa State University, Ames, IA 50011,United States; Department of Biomedical Sciences, Iowa State University, Ames, IA 50011,United States; Department of Biomedical Sciences, Iowa State University, Ames, IA 50011,United States; Department of Biomedical Sciences, Iowa State University, Ames, IA 50011,United States; Department of Biomedical Sciences, Iowa State University, Ames, IA 50011,United States

## Abstract

Nusinersen, an antisense oligonucleotide (ASO) encompassing a phosphorothioate backbone and 2′-*O*-methoxyethyl (MOE) modifications, is commonly used for the treatment of spinal muscular atrophy (SMA), the leading genetic cause of infant mortality. Nusinersen acts through prevention of skipping of exon 7 of *Survival Motor Neuron 2* (*SMN2*) by sequestering Intronic Splicing Silencer N1 (ISS-N1), located within *SMN2* intron 7. Here, we report transcriptome-wide perturbations triggered by ISS-N1-targeting ASOs incorporating diverse modifications, including F18MOE, an 18mer ASO with identical sequence and chemical composition to that of nusinersen. Among cellular processes most impacted by F18MOE were cell cycle, cell growth, cell signaling, and maintenance of the cytoskeleton, chromosomes, and organelles. We demonstrate sequence-dependent and MOE modification-specific off-target effects of F18MOE on transcription and splicing. Owing to unique tolerance for mismatch base pairing with exonic targets, F18MOE triggered skipping of multiple exons, supporting the unexpected role of ISS-N1-like sequences as exonic splicing enhancers. We show that shortening of an ASO suppresses its effect on off-target splicing. Further, we demonstrate using ASOs of mixed chemistry that different MOE-modified regions drive the effect of F18MOE on off-target splicing of different exons. Our findings are instructive in designing future ASO-based therapies and for uncovering novel splicing *cis-*elements.

## Graphical Abstract

**Graphical Abstract:**
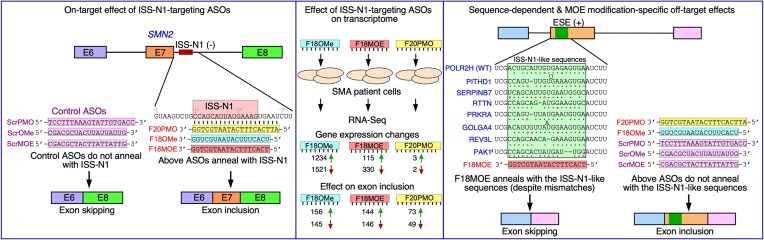

## Introduction

Spinal muscular atrophy (SMA) is a leading genetic disease affecting infants and children caused by deletions of or mutations in the *Survival Motor Neuron 1* (*SMN1*) gene that codes for the multifunctional SMN protein [1, 2]. Humans carry a second copy of the *SMN* gene, *SMN2*, which cannot fully compensate for the loss of *SMN1* due to predominant skipping of exon 7 [3, 4]. Several *cis-*elements and trans-acting factors have been implicated in regulation of splicing of *SMN1/2* exon 7 [5–7]. Correction of *SMN2* exon 7 splicing employing an antisense oligonucleotide (ASO) or a small molecule is a proven strategy for SMA therapy [8]. The discovery of intronic splicing silencer N1 (ISS-N1) within *SMN1/2* intron 7 led to the development of an ASO-based therapy for SMA [9, 10]. The desirable features of ISS-N1 as an ASO target include its relatively large size and uniqueness within the human genome, its location within the intron versus exons, its structural accessibility, and the strong inhibitory effect it exerts on splicing [9, 11, 12]. As a result, the first ISS-N1-targeting ASO encompassing phosphorothioate (PS) backbone and 2′-*O*-methyl (OMe) modification fully restored *SMN2* exon 7 inclusion at concentrations as low as 5 nM [9]. Subsequently, an ISS-N1-targeting ASO encompassing PS backbone and 2′-*O*-methoxyethyl (MOE) modification showed unprecedented therapeutic efficacy *in vivo*, substantially extending the life of SMA mice [13]. Similar *in vivo* efficacies were captured with ISS-N1-targeting phosphorodiamidate morpholino oligonucleotides (PMOs) [14, 15]. Nusinersen (Spinraza^™^), an ISS-N1-targeting ASO encompassing PS backbone and MOE modification became the first FDA-approved drug for the treatment of SMA [10, 16]. Later, gene replacement and a splice-switching small molecule, risdiplam, were approved as therapies for SMA [17, 18]. However, all three available SMA therapies have limitations as patients remain wheelchair bound even after several years of treatment [19]. Some of these limitations may stem from the perturbations of the transcriptome caused by the high concentrations of therapeutic molecules [20–22].

Modulation of splicing employing an ASO-based approach is an area of growing therapeutic interest [23]. In order to confer stability and nuclease resistance *in vivo*, the majority of ASOs are chemically modified at the sugar moiety, including OMe, MOE, and locked nucleic acid (LNA) modifications [24]. Most modified ASOs also incorporate PS backbones to confer nuclease resistance as well as an improved pharmacodynamic profile [25]. Other modifications, such as PMOs, substitute the negatively charged backbone with a neutral one [24]. Critical to the long-term success of an ASO-based therapy, it is imperative that the off-target effects linked to the ASO sequence and/or ASO modifications are appropriately investigated. While a hybridization-dependent off-target effect is exerted through direct base pairing of the ASO to a sequence other than the intended target, hybridization-independent off-target effect is likely realized through interactions of cellular proteins with the ASO. Owing to low tolerance for mismatch base pairing, shorter ASOs tend to have fewer hybridization-dependent off-target effects than the longer ASOs sharing the same target [26–28]. Of note, while all hybridization-dependent effects are expected to be sequence-specific, not all sequence-specific events rely on hybridization, as certain ASO sequences can associate with specific factors with or without interacting with a target sequence [29]. The PS backbone is known to have hybridization-independent effects as it interacts with cellular proteins, often sequestering them in nuclear aggregates that disrupt cellular function [25, 29, 30]. Numerous studies utilizing diverse chemistries, including PS/OMe, PS/MOE, PMO, and LNA have been carried out using ISS-N1 as a target, making it the single most studied ASO target sequence [9, 11, 14, 15, 31]. Recent investigations have shown that ISS-N1-targeting ASOs encompassing PS/OMe modifications cause massive perturbation of the transcriptome of fibroblasts [20, 29]. However, a similar study with an ISS-N1-targeting ASO encompassing PS/MOE modifications, including nusinersen itself has not yet been done. It is also not well studied how the chemistry of a therapeutic ASO impacts the hybridization-dependent off-target effects on splicing due to chemistry-specific differences in tolerance for mismatch base pairing.

Here, we report the findings of a systematic study in which we compared the chemistry-associated and sequence-dependent off-target effects of three ISS-N1-targeting ASOs encompassing PS/OMe, PS/MOE, and PMO modifications. Our results revealed transcriptome-wide changes uniquely triggered by specific ASO modifications. In particular, MOE modification affected expression of several genes and caused hybridization-dependent off-target perturbations on splicing of multiple exons. Our unexpected findings uncover that when present within exons, ISS-N1-like sequences can play a positive role in splicing regulation. Employing minigenes, we validate our findings and demonstrate that the chemistry-specific ability of the MOE ASO to induce skipping of off-target exons is mainly due to tolerance for mismatch base pairing with ISS-N1-like motifs located within the exonic sequences. We show that shortening the ISS-N1-targeting MOE ASO from 18 to 14 or 10 nucleotides (nt) reduces or eliminates the MOE-associated off-target effects on skipping of exons while retaining the on-target effect on inclusion of *SMN2* exon 7. Using ISS-N1-targeting ASOs with mixed modifications, we show that specific positions within the target sequence are critical determinants of the MOE-associated off-target effects. Findings presented here offer novel insights into designing and evaluating future ASO-based therapies, as well as for uncovering novel splicing *cis-*elements.

## Materials and methods

### Cell culture and nucleofection

All cell culture reagents and media were obtained from Life Technologies unless otherwise specified. GM03813 SMA patient fibroblasts were obtained from Coriell Institute of Medical Research and were grown in minimum essential medium (MEM) (Life Technologies #10 370) supplemented with 1× GlutaMAX and 15% fetal bovine serum (FBS). HeLa and HEK293 (HEK) cells were obtained from American Type Culture Collection and were grown in Dulbecco’s Modified Eagles Medium (DMEM) supplemented with 10% FBS. OMe and MOE ASOs were obtained from IDT. PMO ASOs were obtained from GeneTools, LLC. Nusinersen was obtained from MedChemExpress. Specification sheets or mass spectrometry quality control data are included for all ASOs used for RNA-seq experiment and for nusinersen (Supplementary Fig. S1). To nucleofect GM03813 cells, cells were trypsinized, counted, and 2.4 × 10^5^ cells per sample were electroporated in the presence of the desired concentration of ASOs using Lonza P2 Primary Cell 4D-Nucleofector X kit (16-well strip format) in an Amaxa 4D-Nucleofector X using the “EN-150” electroporation protocol. After electroporation, cells were plated in six-well plates, and 6–8 h later the media was replaced with fresh media. For HEK cell nucleofection, 4 × 10^5^ cells per sample were electroporated in the presence of 50 ng plasmid DNA and 6 µM ASOs using Lonza SF cell line kit and plated in 24-well plates. Cells were collected in TRIzol reagent (Life Technologies) 24 h after nucleofection for RNA isolation.

### RNA isolation and RT-PCR

RNA was isolated using TRIzol reagent (Life Technologies) following the manufacturer’s instructions. After isolation, RNA was treated with RQ1 RNase-free DNase (Promega) to remove contaminating DNA, following the manufacturer’s instructions. RNA was then re-purified by phenol:chloroform extraction and ethanol precipitation. To generate complementary DNA (cDNA), reverse transcription (RT) was carried out in 5 µl reactions containing 0.5 µg RNA using Superscript III reverse transcriptase (Life Technologies) following the manufacturer’s instructions. To generate cDNA, RT reactions were primed with random primers (Promega). PCR was carried out using Taq polymerase (NEB) following the manufacturer’s instructions. All primers are listed in Supplementary Table S1. Splice isoforms were quantified by densitometric quantification using ImageJ software. For each isoform, percentage inclusion was calculated by dividing the intensity of its band by the total signal in each lane. Novel isoforms were identified by cloning and Sanger sequencing. Accession numbers for sequence data of skipped isoforms are given in Supplementary Table S2. Quantitative PCR (qPCR) was carried out using PowerUp SYBR green master mix (Life Technologies). Each 10 µl reaction contained 3 µl of 1:40 diluted cDNA (equivalent to 75 ng RNA) and 0.6 µM of each primer. Relative quantification was calculated using the ∆∆Ct method using *OAZ1* transcript as the normalizing assay.

### Library generation and RNA-seq

RNA-Seq library generation and mapping were performed in triplicate. To confirm RNA integrity, TRIzol-isolated total RNA was characterized using an Agilent Bioanalyzer on an RNA nano chip (RIN ≥ 8). One microgram of total RNA was then subjected to ribosomal RNA (rRNA) depletion using the NEBNext rRNA depletion kit v2 (Human/Mouse/Rat). Libraries were generated from rRNA-depleted RNA using the NEBNext Ultra II directional RNA library prep kit for Illumina. Libraries were barcoded for multiplexing using NEBNext Dual Index oligos for Illumina. Size distribution of libraries was determined using an Agilent Bioanalyzer DNA 1000 chip and quantified using a Qubit fluorimeter. Libraries were pooled together and sequenced on an Illumina Novaseq 6000 using an S23 flow cell following a 100-cycle, paired-end protocol. Reads from RNA-Seq were mapped to the human reference genome build GRCh38 using HISAT2 [32]. One untreated sample had a poor mapping rate and thus was excluded from further analyses. For differential expression, mapped reads were assigned to genes according to the Gencode v33 human transcriptome annotation [33] using the featureCounts script from the Subread software package [34]. Differential expression was estimated using the DESeq2 R package [35]. Over-representation analysis (ORA) was performed using the WebGestalt online server (www.webgestalt.org). Transcription factors with binding sites enriched in differentially expressed genes were identified using ChEA3 [36]. To identify differentially affected alternative splicing events, mapped reads were analyzed by rMATS [37]. After initial identification, significant events were subjected to the following filtering criteria: false discovery rate (FDR) < 0.05, 10 or more average junction reads supporting each isoform in at least one sample group, and a change in percentage spliced in (PSI) values of at least 0.1. Putative RNA-binding protein interaction sites were identified using DeepCLIP [38]. RNA-Seq data is publicly available from NCBI’s Gene Expression Omnibus, accession number GSE307431.

### Minigene generation and transfection

The pSMN2 minigene is the same as previously reported pSMN2∆I6 minigene [39]. The pPOLR2H minigene was generated by PCR amplification of the full-length sequence from the start codon of *POLR2H* to the end of exon 3 from genomic DNA isolated from HeLa cells. The PCR product was then cloned into the MCS of pCI mammalian expression vector. Mutations and deletions were introduced using two-step PCR. Hybrid minigenes were generated as described previously [21]. Minigenes were confirmed by Sanger sequencing and sequences were uploaded to NCBI Genbank (Supplementary Table S2). Transfection was carried out as follows: 100 nM of ASO was transfected with or without 0.05 µg of minigenes by reverse transfection of 4 × 10^5^ HEK293 cells or 2.4 × 10^5^ HeLa cells using Lipofectamine 2000 following the manufacturer’s recommendations. Six hours after transfection, media containing transfection complexes was replaced with fresh media. Twenty-four hours after transfection, cells were collected for RNA isolation by TRIzol reagent. Identities of spliced products were confirmed by cloning and Sanger sequencing.

### Depletion of hnRNPA1 and hnRNPA2B1

To knock down hnRNPA1 and/or hnRNPA2B1 expression, 1.25 × 10^6^ HeLa cells were reverse transfected using Lipofectamine 2000 in six-well plates with 50 nM of individual short interfering RNA (siRNA) against the indicated gene or 25 nM each of siRNA against hnRNPA1 and hnRNPA2B1 for co-depletion. Thirty hours after transfection, cells were trypsinized, counted, and plated in 24-well (1.1 × 10^5^ cells) or 6-well (4.4 × 10^5^ cells) plates. The next day (48 h after initial transfection), cells were transfected with 20 nM of the indicated siRNA (10 nM each for double knockdown), 80 nM of ASO, and 50 ng of minigene (for 24-well plates). For six-well plates, minigene was omitted. Cells in 24-well plates were collected in TRIzol for RNA isolation 24 h later (72 h after initial transfection). Cells in six-well plates were collected by scraping in ice-cold phosphate buffered saline (PBS) and processed for protein lysate generation and western blotting.

### Protein lysate preparation and western blotting

Cells in PBS were collected by centrifugation for one minute at 3500 × *g* at 4°C. Supernatant was aspirated and cells were resuspended in 30–60 µl of Radioimmunoprecipitation Assay (RIPA) buffer (Boston Bioproducts) supplemented with 1× HALT protease and phosphatase inhibitor cocktail (Thermo Fisher). To ensure complete lysis, cells were incubated for 20 min on ice. Lysates were centrifuged for 15 min at 12 000 × *g* at 4°C, and supernatant was transferred to a new tube. Protein concentration was measured using Bio-Rad Protein Assay dye reagent following the manufacturer’s instructions. Twenty micrograms of each protein sample was separated on a 10% sodium dodecyl sulfate polyacrylamde gel electrophoresis (SDS–PAGE) gel and transferred to polyvinylidene difluoride (PVDF) membranes using the Bio-Rad Trans-Blot Turbo Transfer system. Membranes were blocked with 5% nonfat milk for one hour at room termperature in 1× Tris-buffered saline with 0.05% Tween-20 (TBST) with gentle agitation and probed with 1:5000 mouse anti-hnRNPA1 (Abcam ab5832, clone 9H10) or 1:1000 mouse anti-hnRNPA2B1 (Abcam ab6102, clone DP3B3) overnight at 4°C with gentle agitation. After primary antibody incubation, blots were washed with 1× TBST three times for at least 10 min and incubated with 1:5000 goat anti-mouse antibody conjugated to horseradish peroxidase (HRP) (Jackson Immunoresearch 115-035-003) for 1 h at room temperature with gentle agitation. Blots were then washed with 1× TBST three times for at least 10 min. Blots were developed using Clarity western blot ECL substrate (Bio-Rad) or SuperSignal West Femto Maximum Sensitivity substrate (Thermo Fisher) and scanned using a UVP BioSpectrum AC Imaging System (UVP). To confirm loading, blots were stripped using Restore western blot stripping buffer (Thermo Fisher) and reprobed using 1:4000 mouse monoclonal anti-Tubulin (Sigma-Aldrich T6199) primary antibody for 1 h at room temperature. Secondary antibody incubation and development were carried out as described above.

## Results

### Transcriptome-wide effect of ISS-N1 targeting ASOs encompassing different chemistries

**Figure 1.**
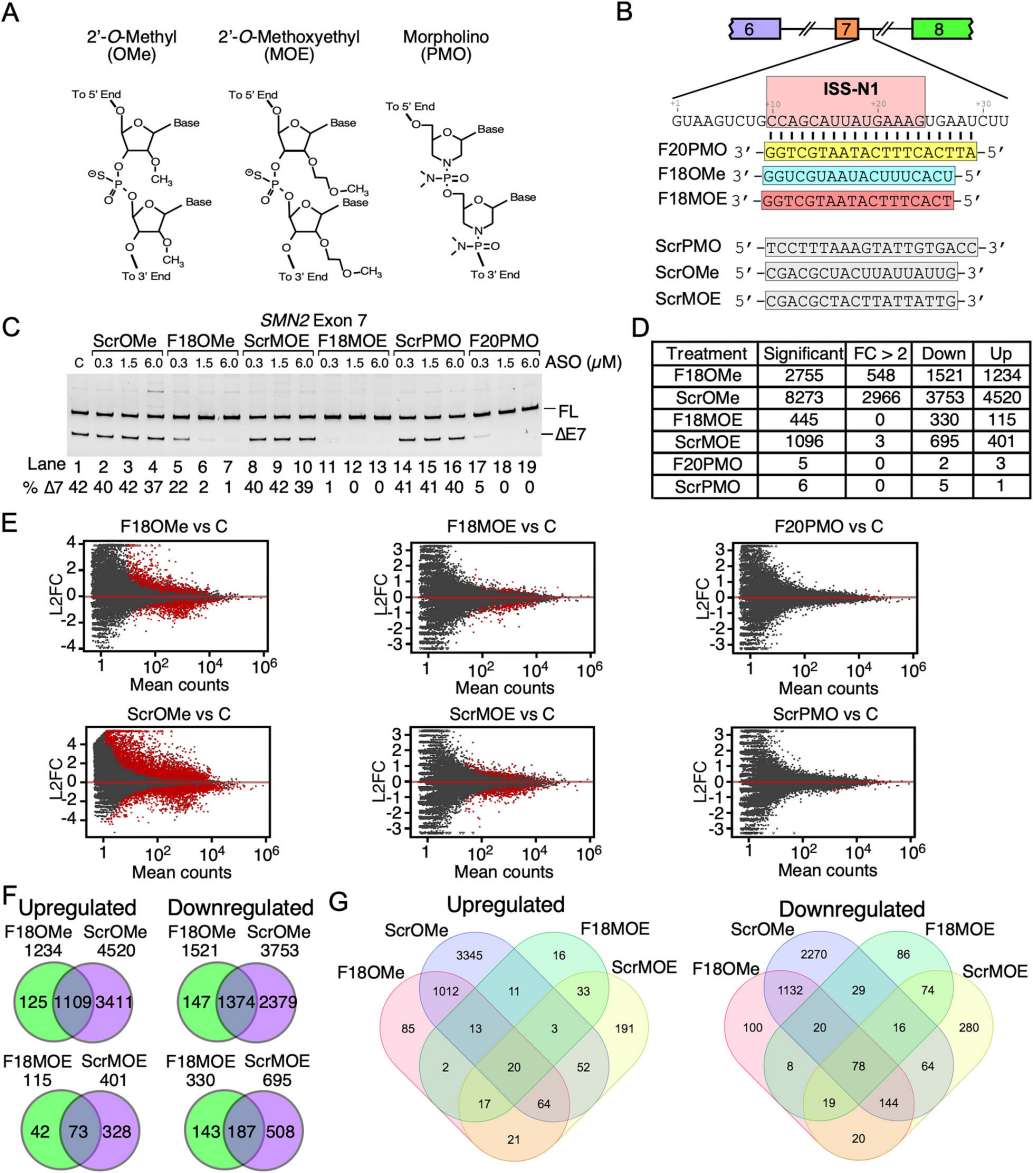
RNA-Seq analysis of SMA patient fibroblasts treated with three chemistries of ISS-N1-targeting ASOs. (**A**) Partial chemical structures of three types of ISS-N1-targeting ASOs used to correct *SMN2* exon 7 splicing in this study. (**B**) Annealing locations and sequences of the three ASOs and nontargeting (Scr) controls. An overview of the *SMN2* exon 7 region is given. Exons are depicted as colored boxes, introns as broken lines. The sequence of the first 32 bases of intron 7 are given. ISS-N1 is boxed in pink, with ASOs shown below. Base pairs between ASOs and the ISS-N1 region are indicated with black lines. (**C**) Representative gel image depicting splicing pattern of *SMN2* exon 7 in SMA patient fibroblasts upon treatment with the indicated ASOs. Treatments are indicated at the top of the gel. “C” indicates cells without any ASO treatment. “FL” indicates exon 7 inclusion, “∆E7” indicates exon 7 skipping. Percentage skipping of exon 7 is indicated at the bottom of the gel. (**D**) Summary of differential expression of genes upon treatment with the indicated ASOs. All comparisons were made relative to untreated cells. “Significant” indicates all genes with significantly changed expression (Benjamini and Hochberg adjusted *P* value (adj. *P*) < 0.05). “FC > 2” indicates genes with more than 2-fold change in expression levels. “Down” – downregulated, “Up” – upregulated. (**E**) MA plots visualizing differential expression of genes upon treatment with the indicated ASOs. Each comparison is indicated above the plot. *Y*-axis represents log_2_-transformed fold change (L2FC) of gene expression. *X*-axis depicts mean counts of gene expression across all samples. Each dot represents one gene, red dots indicate significant genes (adj. *P* < 0.05). (**F**) Venn diagrams depicting the overlap between genes upregulated (left panels) and downregulated (right panels) upon treatment with ISS-N1-targeting ASOs or their respective controls. The treatment type and total upregulated/downregulated genes are indicated at the top of each diagram. (**G**) Venn diagrams depicting the overlap between genes upregulated (left panel) and downregulated (right panel) by all four OMe-modified and MOE-modified ASOs.

We employed GM03813 SMA patient fibroblasts to compare the transcriptome-wide effects of three ISS-N1-targeting ASOs: F18OMe, F18MOE, and F20PMO (Fig. 1A and B). F18OMe encompasses OMe modifications at the 2′ position of every ribose sugar of the RNA backbone, while F18MOE encompasses MOE modifications at the same position (Fig. 1A). Both F18OMe and F18MOE are 18mer ASOs and incorporate PS linkages replacing the phosphodiester bond. F18OMe and F18MOE harbor U and T residues, respectively, that base pair with A residues of the target sequence, and F18MOE incorporates methylcytosine in place of cytosine. F18MOE is identical to nusinersen, an approved drug in many countries, including USA, Europe, and Japan, for the treatment of SMA [10]. F20PMO is a 20mer PMO comprised of neutral backbone distinct from F18OMe and F18MOE (Fig. 1A) [14]. Of note, we used the 20mer PMO due to previous reports of longer sequence requirement for the best annealing efficacy using this chemistry [40]. F20PMO represents the shortest PMO known to correct *SMN2* exon 7 splicing in SMA model mice [14]. As controls, we used scrambled ASOs (ScrOMe, ScrMOE, and ScrPMO) encompassing identical chemistries to those of the corresponding experimental ASOs but with randomized base order (Fig. 1B). We began our study with a pilot nucleofection experiment employing different concentrations of the ASOs and captured >50% correction of *SMN2* exon 7 splicing at 0.3 µM, which we chose as the lowest ASO concentration (Fig. 1C). The highest chosen concentrations of 6 µM of ISS-N1-targeting ASOs produced full correction of *SMN2* exon 7 splicing, while the scrambled controls had no discernable effect (Fig. 1C and Supplementary Fig. S2). In order to capture the transcriptome-wide off-target effects, we used 6 µM ASO concentration as this concentration falls within the feasible clinical dose and is routinely used for oligonucleotides in nucleofection experiments [41–43].

Using RNA-Seq profiling, we captured varying degrees of perturbations of the transcriptome by ISS-N1-targeting ASOs. F18OMe affected the expression of 2755 genes (out of 27 369 expressed genes that were analyzed), or about 10% of the cellular transcriptome (Fig. 1D and E). 548 of these genes were affected >2-fold, and there were slightly more downregulated genes than upregulated. ScrOMe affected the expression of 8273 genes, ∼3 times more than F18OMe. Of the 2966 genes affected >2-fold by ScrOMe, a relatively higher number were upregulated than downregulated (Fig. 1D and E). We found a strong overlap between targets of F18OMe and ScrOMe, supporting a high level of hybridization-independent off-target effects of ASOs encompassing OMe modifications (Fig. 1F). F18MOE affected the expression of 445 genes, none of which were altered >2-fold. Twice as many genes were downregulated than upregulated by F18MOE (Fig. 1D and E). While ScrMOE altered expression of 1096 genes, expression of only three genes was affected >2-fold. As observed in case of F18MOE, most affected targets of ScrMOE were downregulated. F18MOE and ScrMOE shared 63% and 57% of the upregulated and downregulated targets, respectively (Fig. 1F). We also captured an overlap of ten targets, two upregulated and eight downregulated, between F18OMe and F18MOE but not by their respective scrambled controls, underscoring a sequence-specific effects by these two ISS-N1-targeting ASOs encompassing PS backbone. Additional overlaps between F18OMe and F18MOE were shared by one or both scrambled ASOs, supporting hybridization-independent off-target effects of the PS backbone (Fig. 1G). F20PMO had the fewest off-target effects as only 5 genes were impacted, while ScrPMO affected 6 genes (Fig. 1D and E). None of the PMO targets were affected >2-fold and we could not independently validate the changes by qPCR, underscoring negligible levels of PMO-induced off-target effects (Fig. 1D and E, and Supplementary Fig. S2).

We performed ORA of the genes that were upregulated and downregulated by F18Ome and F18MOE. Since F20PMO affected very few genes, such an analysis was not possible for its targets. Genes that were upregulated by F18OMe were enriched for gene ontology (GO) terms related to transcriptional repression (DNA-binding transcriptional repressor, transcription corepressor activity), cell signaling (cellular response to external stimulus, protein serine/threonine kinase activity, stress-activated protein kinase signaling, regulation of protein serine/threonine kinase, response to interleukin-1), and differentiation/growth arrest (fat cell differentiation, neuron projection guidance, cell cycle arrest) (Supplementary Fig. S3A). Consistent with the results of GO term enrichment, the functional Kyoto Encyclopedia of Genes and Genomes (KEGG) pathways that were enriched among genes upregulated by F18OMe were mostly involved in various cancers, indicating a mis-regulation of cell division and differentiation, as well as two signaling pathways (MAPK signaling pathway and FoxO signaling pathway) (Supplementary Fig. S3A). The GO terms and KEGG pathways that were enriched among genes downregulated by F18OMe were almost all related to either DNA or RNA metabolism or cell division, further supporting that F18OMe has a negative impact on genes involved in cell growth and/or division (Supplementary Fig. S3B). We also performed ORA for specific chromosomal regions. There was striking enrichment for two neighboring genomic regions in chromosome 6, chr6p22.1 and chr6p22.2 (Supplementary Fig. S3B). These regions contain many genes encoding histone proteins that were downregulated by F18OMe. Unlike in the case of F18OMe, we did not observe a significant enrichment of GO terms or KEGG pathways associated with genes that were upregulated by F18MOE. However, we captured significant enrichment of F18MOE-induced upregulated genes in the chr11q22.2 region (Supplementary Fig. S3C). This region contained three upregulated genes coding for matrix metalloproteases (MMPs), MMP1, MMP3, and MMP10. F18MOE-induced downregulated genes were associated with the enrichment of GO terms related to cell division (Supplementary Fig. S3D). These results supported that similar to F18OMe, F18MOE may delay cell cycle and/or cause cell growth arrest.

To identify potential mechanisms behind ASO-mediated changes in gene expression, we analyzed downregulated and upregulated genes for common transcription factor binding sites using ChEA3 [36] (Supplementary Table S3). Genes downregulated by F18MOE, ScrMOE, F18OMe, and ScrMOE were all highly enriched for binding sites for FOXM1 and CENPA (Supplementary Table S3A–D). In addition, binding sites for E2F7, E2F1, and MYBL2 were enriched among genes downregulated in 3 out of 4 categories. Binding sites for PRMT3, ZNF146, PA2G4, and ZNF770 were enriched among genes upregulated by both F18MOE and F18OMe but not either scrambled control, suggesting some sequence-specific, chemistry-independent effects among upregulated genes (Supplementary Table S3E–H).

### Validation of gene expression changes triggered by ISS-N1-targeting ASOs

**Figure 2.**
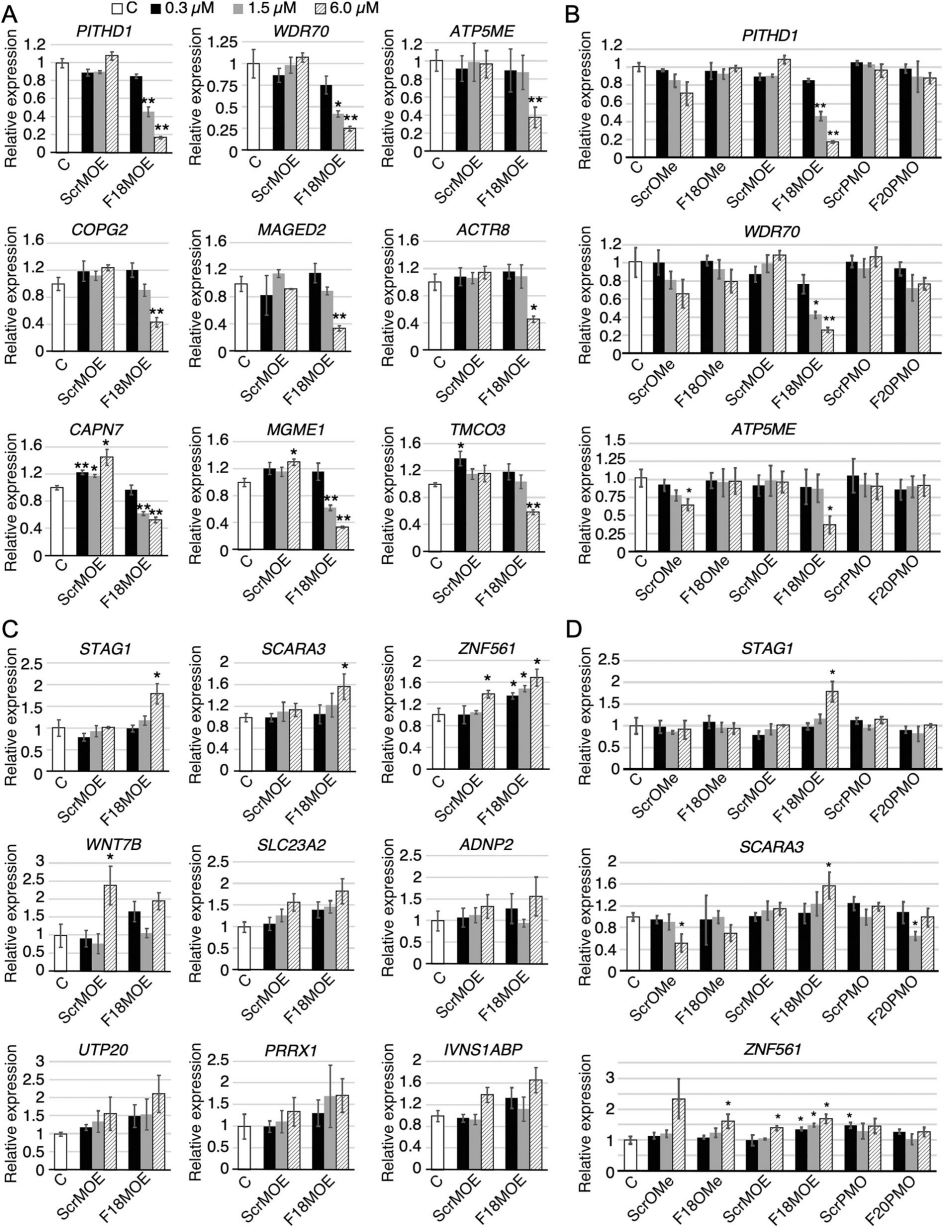
Validation of differential gene expression triggered by ISS-N1-targeting ASOs. (**A**) Transcript levels of genes predicted by RNA-Seq to be downregulated by F18MOE in a sequence-specific manner, as measured by qPCR. ASO identities are indicated at the bottom (x axis). “C” indicates cells without any ASO treatment. Error bars represent standard error of the mean (SEM). * - *P *< 0.05, ** - *P *< 0.01. (**B**) Transcript levels of three candidate genes downregulated by F18MOE upon treatment with varying concentrations of all six ASOs, as measured by qPCR. Coloring and labeling are the same as in (**A**). (**C**) Transcript levels of genes predicted by RNA-Seq to be upregulated by F18MOE in a sequence-specific manner, as measured by qPCR. Coloring and labeling are the same as in (**A**). (**D**) Transcript levels of three candidate genes upregulated by F18MOE upon treatment with varying concentrations of all six ASOs, as measured by qPCR. Coloring and labeling are the same as in (**A**).

We used qPCR to independently validate the ISS-N1-targeting ASO-induced gene expression changes captured by RNA-Seq. We performed these experiments at three ASO concentrations: 0.3, 1.5, and 6 µM. We specifically focused on F18MOE due to its identical chemical composition to nusinersen and selected candidates with diverse functions (Supplementary Table S4). We first validated sequence-specific effects of F18MOE on nine genes predicted by RNA-Seq to be strongly downregulated, none of which were similarly affected by ScrMOE (Fig. 2A). Results of qPCR confirmed significant downregulation of all nine genes by 6 µM of F18MOE. Four of these genes, *PITHD1, WDR70, MGME1*, and *CAPN7*, were also significantly reduced by 1.5 µM of F18MOE. To confirm that the effect of F18MOE-induced downregulation of the above genes was also chemistry-specific, we compared the expressions of top three genes, *PITHD1, WDR70*, and *ATP5ME*, in GM03813 cells treated with three concentrations (0.3, 1.5, and 6 µM) of F18OMe, F18MOE, F20PMO, and their respective scrambled controls (Fig. 2B). *PITHD1* codes for PITH domain-containing protein 1 that interacts with the proteasome and may play a role in protein turnover [44]. Expression of *PITHD1* was decreased by F18MOE in a concentration-dependent manner, with 1.5 and 6 µM concentrations reducing expression >2-fold and >5-fold, respectively (Fig. 2B). Other ASOs had no effect, even at their highest concentration (Fig. 2B). Of note, downregulation of *PITHD1* by F18MOE could be due to nonsense-mediated decay (NMD), as analysis of our RNA-Seq data and subsequent validation confirmed F18MOE-induced skipping of *PITHD1* exon 2, creating a frameshift in the coding sequence (described later). *WDR70* codes for a WD repeat-containing protein that plays an essential role in DNA damage repair through regulation of histone H2B lysine ubiquitination [45]. F18MOE treatment downregulated *WDR70* expression in a concentration-dependent manner, with 1.5 and 6 µM concentrations reducing expressions > 2- and ∼4-fold, respectively (Fig. 2B). Here again, other ASOs had no effect on expression of *WDR70*, even at their highest concentration. *ATP5ME* (also known as *ATP5I* or *ATP5K*) encodes a membrane-spanning subunit of the mitochondrial ATP synthase machinery [46]. The highest concentration (6 µM) of F18MOE caused about ∼2-fold downregulation of *ATP5ME*, whereas ScrMOE had no effect (Fig. 2B). The highest concentration of ScrOMe, but not F18OMe, also produced a small but noticeable downregulation of *ATP5ME* (Fig. 2B). Overall, our results confirmed the sequence-dependent and chemistry-specific effect of F18MOE on downregulation of *PITHD1, WDR70* and *ATP5ME* (Fig. 2B).

**Figure 3.**
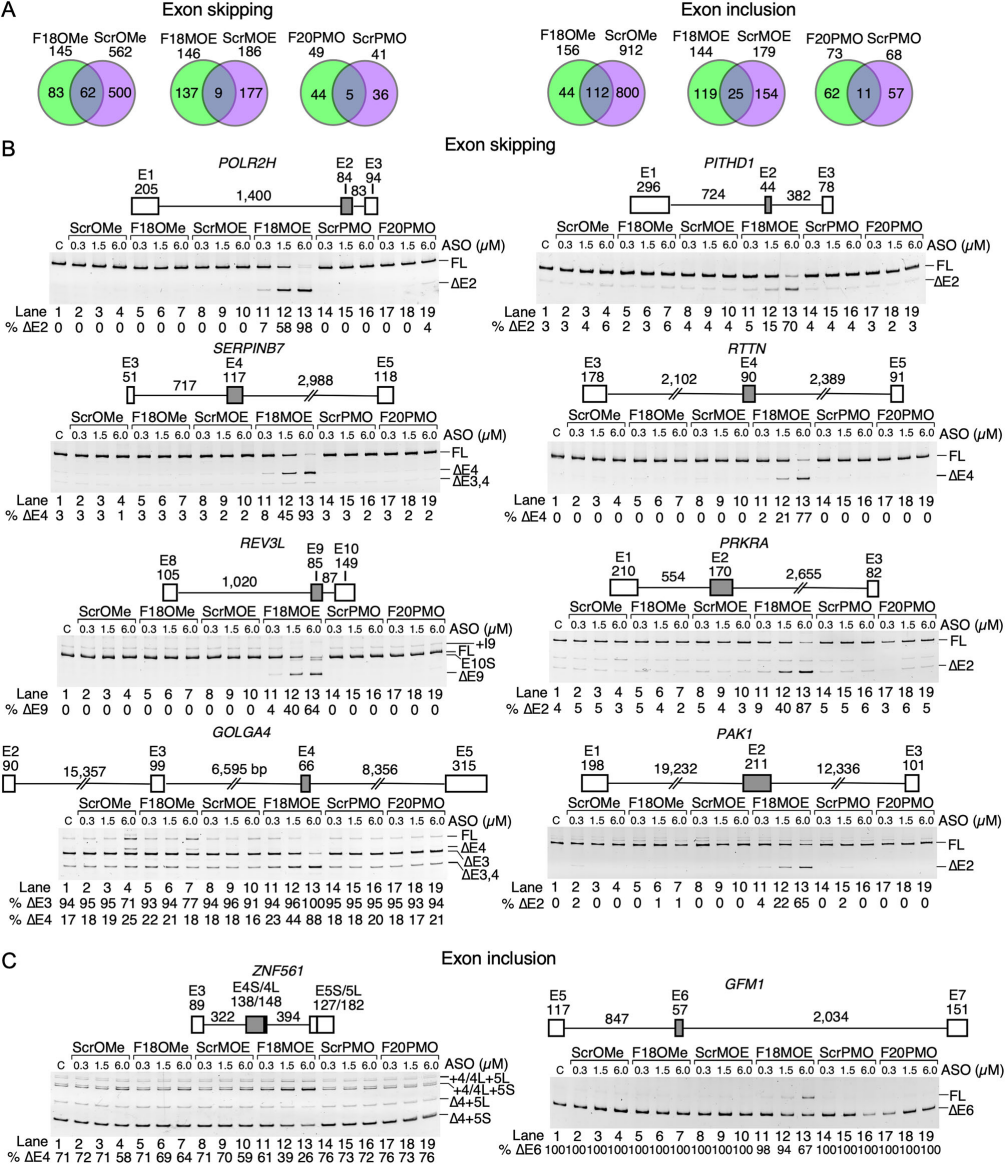
Alternative splicing events affected by ISS-N1-targeting ASOs. (**A**) Venn diagrams depicting the overlap between exon skipping events and inclusion events by treatment with ISS-N1-targeting ASOs and scrambled control of each ASO chemistry in SMA fibroblasts. The treatment type and total skipped/included exons are indicated at the top of each diagram. (**B**) Genomic overview and splicing pattern of eight exons predicted by RNA-Seq to undergo increased skipping upon treatment with F18MOE. Exons are depicted as boxes, introns as lines/broken lines. Exon and intron sizes are indicated. Alternatively spliced exons are colored grey. Gels are labeled similarly as in Fig. 1C. (**C**) Splicing pattern of two exons predicted by RNA-Seq to undergo increased inclusion after treatment by F18MOE.

We performed qPCR to validate nine randomly selected genes predicted by RNA-Seq to be upregulated by F18MOE. Only three of these genes, *STAG1, SCARA3*, and *ZNF561*, were confirmed by qPCR to undergo significant upregulation by F18MOE (Fig. 2C). *STAG1* codes for a component of cohesin complex that connects sister chromatids [47]. While 6 µM of F18MOE increased *STAG1* expression ∼1.7-fold, lower concentrations of F18MOE and all concentrations of ScrMOE had no significant effects (Fig. 3B). *SCARA3* (also known as *CSR*) codes for a member of the scavenger receptor-class A family that is induced by and mitigates oxidative stress [48]. Interestingly, SCARA3 is reportedly involved in the uptake of the cell-penetrating peptide-conjugated ASOs and also colocalizes with the naked ASOs [49]. While 6 µM concertation of F18MOE increased *SCARA3* expression ∼1.6-fold, lower concentrations and ScrMOE had no effect (Fig. 3B). *ZNF561* codes for zinc-finger protein 561, aberrant expression of which is associated with colorectal cancer [50]. While all concentrations of F18MOE increased expression of *ZNF561*, the effect was most pronounced at 6 µM (Fig. 2C). Although not captured by RNA-Seq, highest concentration of ScrMOE also increased expression of *ZNF561* (Fig. 2C). To test whether the effect of F18MOE-induced upregulation of *STAG1, SCARA3*, and *ZNF561* was chemistry-specific, we compared their expressions in GM03813 cells treated with three concentrations (0.3 µM, 1.5 µM, and 6 µM) of F18OMe, F18MOE, F20PMO and their respective controls (Fig. 2D). Our results confirmed sequence-dependent and chemistry-specific effect of F18MOE on upregulation of *STAG1, SCARA3* and *ZNF561* (Fig. 2D). However, the highest concentrations of F18OMe and ScrOMe also triggered upregulation of *ZNF561*, although difference was not statistically significant in case of ScrOMe (Fig. 2D).

Although F18MOE was designed to be chemically identical to nusinersen, there remains a possibility that differences in manufacturing and/or purity are driving some or all of its off-target effects. Therefore, we obtained commercially available nusinersen and transfected HeLa cells with 100 nM ScrMOE, F18MOE, or nusinersen for 24 hours. Confirming the similarity between F18MOE and nusinersen, six out of eight genes downregulated by F18MOE (*PITHD1, WDR70, COPG2, CAPN7, MGME1*, and *TMCO3* were similarly affected by nusinersen (Supplementary Fig. S4A). While nusinersen treatment did not significantly downregulate *ACTR8* expression, expression trended downward and the nusinersen-treated sample was not statistically different from F18MOE. Only *ATP5ME* failed to show any effect of nusinersen at all (Supplementary Fig. S4A). In contrast, none of the genes predicted by RNA-seq to be upregulated in SMA patient fibroblasts were significantly affected in HeLa cells by either ASO, perhaps due to the cell line or method of delivery (Supplementary Fig. S4B).

To evaluate the hybridization-independent off-target effects of MOE ASOs, we performed qPCR validation of the expression of twelve genes predicted by RNA-Seq to be dysregulated by both F18MOE and ScrMOE. Eight of these genes, namely *SLC6A9, PKD1, TNKS1BP1, SUN2, STAT2, JPH2, DDX6* and *ZNF460* were downregulated, whereas four of these genes, including *PRSS3, IRAK1, PPAT* and *TMEM97* were upregulated. Among the downregulated genes, qPCR only confirmed the expected trend for three genes out of eight, and only *SCL6A9* was significantly altered in both F18MOE and ScrMOE treatment (Supplementary Fig. S5A). All four upregulated genes trended upwards upon ASO treatment, and three (*PRSS3, PPAT*, and *TMEM97*) were significantly altered by both F18MOE and ScrMOE (Supplementary Fig. S5B). Analysis of RNA-Seq revealed expression of genes that were similarly affected by all four ASOs encompassing PS backbone, including F18MOE, ScrMOE, F18OMe and ScrOMe. We validated the expression of eight such genes, four downregulated (*PIMREG, KANK1, SFT2D2* and *KRT34*) and four upregulated (*MMP16, MICAL2, BAALC* and *SERPINB2*). The results of qPCR showed poor consensus with the RNA-Seq predictions among the downregulated genes (Supplementary Fig. S6A). All upregulated genes showed the expected trend in expression, although a significant change was captured in all four ASOs only for *MICAL2* (Supplementary Fig. S6B). F18MOE and ScrMOE but not the OMe-containing ASOs caused significant downregulation and upregulation of *KRT34* and *MMP16*, respectively. F18OMe and ScrOMe, but not the MOE-containing ASOs, caused significant downregulation and upregulation of *PIMREG* and *BAALC*, respectively.

To determine whether the effects of F18MOE on the transcriptome were dependent on the cell type and the method of F18MOE delivery, we treated four cell types (GM03813 SMA patient fibroblasts, HeLa cells, HEK293 cells, and neuron-like SH-SY5Y cells) with 100 nM of F18MOE and ScrMOE for 24 hours by lipofectamine-mediated transfection. We then measured the expression of three downregulated (*PITHD1, WDR70*, and *ATP5ME*) and three upregulated (*STAG1, SCARA3*, and *ZNF561*) genes by qPCR. Confirming the transfection efficiency, all ISS-N1-targeting ASOs restored *SMN2* exon 7 inclusion in all four cell types examined, although the effect was modest in SH-SY5Y cells, likely due to poor transfectability of this cell line (Supplementary Fig. S7). While *PITHD1* and *WDR70* were significantly downregulated by F18MOE in all four cell types, *ATP5ME* was downregulated in GM03813, HEK293, and HeLa cells, but not in SH-SY5Y cells. Of the upregulated genes, *STAG1* showed a significant increase in expression by F18MOE in GM03813 and HeLa cells, but not in HEK293 and SH-SY5Y cells. While statistically significant changes were not captured for *SCARA3* and *ZNF561*, we observed a trend toward upregulation for these genes in GM03813 and HeLa cells. Interestingly, we observed a trend toward downregulation for *SCARA3* in HEK293 and SH-SY5Y cells, and for *ZNF561* in SH-SY5Y cells. Nusinersen has been shown to influence transcription by inducing the inhibitory H3K9me2 mark on nearby chromatin when it binds to RNA or possibly DNA [51]. We examined the gene loci of genes downregulated by F18MOE for potential ASO annealing sites. We did not observe any perfect matches, and allowing for more than two mismatches between the ASO and target sequence resulted in too many potential binding sites to analyze. To determine if F18MOE is influencing gene expression of multiple neighboring genes by altering chromatin structure, we examined the locations of all the genes that we selected for qPCR validation and the expressions of their nearest neighboring genes (Supplementary Fig. S8). Aside from some limited cases, we did not observe any significant enrichment of genes with specific alterations in expression in the immediate vicinity of our candidate genes.

### Differential effects of ISS-N1-targeting ASOs on alternative splicing

We analyzed exon inclusion and skipping events that were triggered by the ISS-N1-targeting ASOs. We identified 156 candidate cassette exons that were predicted to undergo increased inclusion upon treatment with F18OMe (Fig. 3A). More than two-thirds of these events were similarly altered by ScrOMe. Of the 145 exons that were predicted to undergo F18OMe-induced enhanced skipping, 62 of them also underwent skipping by ScrOMe. These results supported a pervasive sequence-independent effect of OMe modifications on both inclusion and skipping events. Our RNA-Seq analysis predicted F18MOE-induced increased inclusion and skipping of 144 and 146 alternatively spliced exons, respectively (Fig. 3A). The majority of the inclusion events were unique to F18MOE, as only 25 events were shared with ScrMOE. Almost all skipping events were specific to F18MOE, as there was very little overlap of skipping events triggered by ScrMOE. These results supported an overall reduced sequence-independent effect of MOE modifications compared to OMe modifications. F20PMO was predicted by RNA-Seq to trigger inclusion and skipping of 73 and 49 alternatively spliced exons, respectively (Fig. 3A). However, upon manual examination of RNA-Seq data, most events turned out to be minor and/or low-confidence, suggesting minimal off-target on splicing by F20PMO.

In order to validate key alternative splicing events, we treated GM03813 cells with low (0.3 µM), intermediate, (1.5 µM), and high (6.0 µM) concentrations of ASOs. We validated the alternative splicing of eight randomly selected exons whose skipping was enhanced by F18MOE: *POLR2H* exon 2, *PITHD1* exon 2, *SERPINB7* exon 4, *RTTN* exon 4, *REV3L* exon 9, *PRKRA* exon 2, *GOLGA4* exon 4, and *PAK1* exon 2. The selected exons were located in diverse chromosomes and exhibited a broad range of sizes (44 to 211 nt), were flanked by introns of varied lengths (83 nt to almost 20 kb) (Fig. 3B). All eight exons are located within the coding regions of their respective proteins. Four (*POLR2H* exon 2, *SERPINB7* exon 4, *RTTN* exon 4, and *GOLGA4* exon 4) skipping events are predicted to preserve the coding frame, while four (*PITHD1* exon 2, *REV3L* exon 9, *PRKRA* exon 2, and *PAK1* exon 2) do not, potentially leading to NMD. Using semi-quantitative PCR, we confirmed a significant increase in skipping (>50%) triggered by treatment with 6 µM of F18MOE in all eight events (Fig. 3B) 1.5 µM also caused a noticeable increase in skipping in all cases as well, although the effect was more varied. Of the exon skipping events that we validated, F18MOE had the strongest impact on *POLR2H* exon 2, with >50% skipping at 1.5 µM and almost complete skipping at 6 µM (Fig. 3B). We did not observe any change in skipping of any of the exons upon treatment with F18OMe or F20PMO or any of the scrambled control ASOs, indicating that all of the events that we validated were both sequence- and chemistry-specific.

We validated seven candidate exons that were predicted by RNA-Seq to undergo increased inclusion in the presence of F18MOE (Fig. 3C and Supplementary Fig. S9). *ZNF561* exon 4 was the most strongly included exon after F18MOE treatment, followed by *GFM1* exon 6 (Fig. 3C). All other exons had mild (<15%) or no increase in inclusion upon F18MOE treatment (Supplementary Fig. S9). *ZNF561* exons 4 and 5 have a complex splicing pattern, with both exons utilizing alternative 3′ss. F18MOE decreased skipping of *ZNF561* exon 4 from 71 percent to 26 percent, without any change in the apparent ratio of isoforms generated by either alternative 3′ss (Fig. 3C). We also noted decreased skipping of *ZNF561* exon 4 at the highest concentrations of ScrOMe, F18OMe, and ScrMOE, suggesting that both ASO chemistry (specifically, the phosphorothioate backbone shared between OMe and MOE chemistries) and sequence play roles in inclusion of *ZNF561* exon 4. We observed an increase in *GFM1* exon 6 inclusion from complete skipping to 67 percent inclusion, but only at the highest concentration of F18MOE (Fig. 3C). Based on these results, it is possible that F18MOE annealing is far more likely to induce exon skipping than exon inclusion.

To rule out that the effects of F18MOE on alternative splicing of off-target exons could be due to manufacturing or purity, we tested the effects of three concentrations of F18MOE and commercially available nusinersen on splicing of *SMN1/2* exon 7 and seven exons that were skipped upon F18MOE treatment in HeLa cells. As expected, *SMN1/2* exon 7 inclusion was fully restored at all three concentrations of both ASOs, while skipping of all of the off-target exons was increased in a concentration-dependent manner (Supplementary Fig. S10). The effectiveness of F18MOE and nusinersen was nearly identical for all exons, suggesting that F18MOE and nusinersen are equivalent in their off-target effects on splicing. Therefore, given that we have also shown that F18MOE is equivalent to nusinersen in its ability to modify gene expression (Supplementary Fig. S4), we conclude that any conclusions drawn using F18MOE can be broadly applicable to nusinersen as well.

In order to better understand the mechanism of sequence- and chemistry-specific off-target effects on splicing, we examined potential annealing positions of the ISS-N1-targeting ASOs to each of the most strongly affected exons and their surrounding intronic areas (Supplementary Fig. S11). All eight of the validated skipped exons had potential annealing sites for F18MOE, although each had at least three mismatches, bulges, or G-T/G-U wobble base pairs. Although the position of the prospective annealing sites was distributed throughout the target exons without any clear pattern, none were located in the flanking introns, suggesting that annealing of the ASO to exonic sequences is much more likely to trigger skipping than annealing to intronic sequences (Supplementary Fig. S11). We used MaxEntScan to evaluate splice site strength of the 3′ss and 5′ss of the skipped exons. We observed no clear connection between splice site strengths and skipping of the candidate exons (Supplementary Fig. S11).

### Tolerance for mismatch base pairings underlie MOE-specific off-target effect

**Figure 4.**
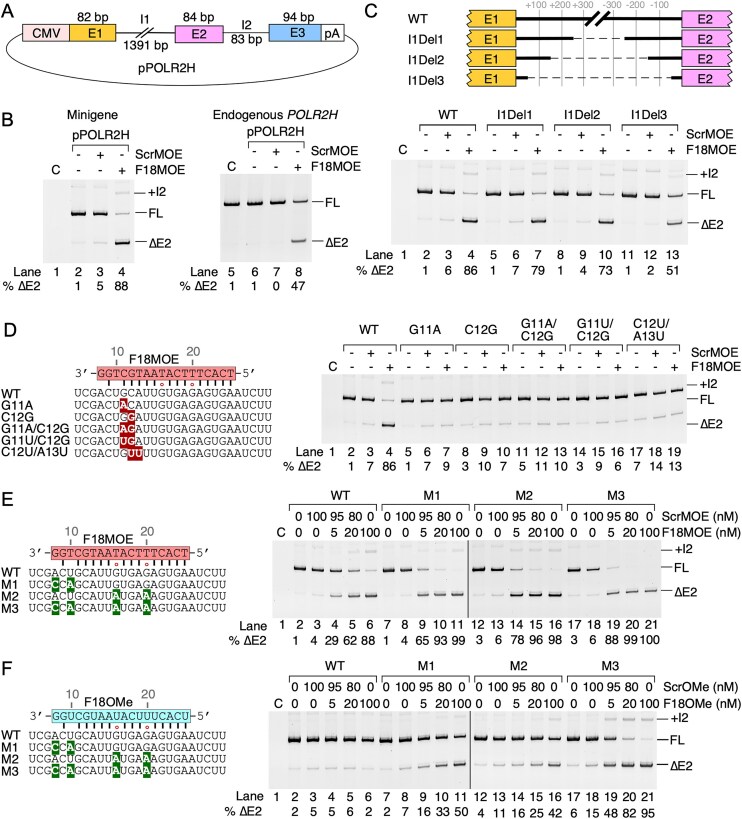
F18MOE induces skipping of *POLR2H* exon 2 by annealing to exonic sequences. (**A**) Overview of pPOLR2H minigene. Exons, CMV promoter, and termination signal are depicted as boxes, introns as lines/broken lines. Sizes of exons and introns are indicated. (**B**) Splicing pattern of transcripts generated from pPOLR2H (left) and endogenous *POLR2H* (right) cotransfected with 100 nM of the indicated ASO. Labeling is similar to Fig. 1C. (**C**) Top panel: Overview of intron 1 deletions in pPOLR2H. Exons are shown as boxes, introns as solid black lines. Deletions are indicated with dashed lines. Numbering is given relative to the closest exon. Lower panel: Splicing pattern of transcripts generated from pPOLR2H carrying intronic deletions cotransfected with 100 nM of the indicated ASO. (**D**) Left panel: Annealing location of F18MOE in *POLR2H* exon 2 and mutated sequences designed to weaken ASO annealing. Canonical base pairs are indicated with black lines. G-U/G-T “wobble” base pairs are indicated with open red circles. Mutated bases are highlighted in red. Right panel: Mutated pPOLR2H cotransfected with 100 nM of the indicated ASOs. (**E**) Left panel: Annealing location of F18MOE in *POLR2H* exon 2 and mutated sequences designed to strengthen ASO annealing. Mutated bases are highlighted in green. Right panel: Splicing pattern of transcripts generated from mutant pPOLR2H cotransfected with increasing amounts of F18MOE (0, 5, 20, and 100 nM). (**F**) Left panel: Annealing location of F18OMe in *POLR2H* exon 2 and mutated sequences designed to strengthen ASO base pairing. Right panel: Splicing pattern of transcripts generated from mutant pPOLR2H cotransfected with increasing amounts of F18OMe (0, 5, 20, and 100 nM).

Next, we generated a *POLR2H* cassette minigene to examine the F18MOE-associated off-target effect on splicing of exon 2. The minigene harbored the genomic sequence spanning from the first exon through the third exon of *POLR2H*, driven by the CMV promoter (Fig. 4A). We cotransfected the minigene with 100 nM of F18MOE or scrambled control into HEK293 cells and examined the splicing pattern of transcripts generated from the minigene. As expected, F18MOE but not the control ASO caused predominant skipping of exon 2, both in transcripts generated from *POLR2H* minigene and endogenous *POLR2H* (Fig. 4B). The effect on endogenous *POLR2H* exon 2 was less pronounced, however, likely due to background of the untransfected cells (Fig. 4B). The majority of the *POLR2H* minigene sequence consists of the ∼1.4 kb long intron 1 (Fig. 4A). To rule out that F18MOE binds to the intronic sequences, we made mutant *POLR2H* minigenes with internal deletions within intron 1, resulting in a truncated intron of ∼500, ∼300, and ∼100 nucleotides (Fig. 4C, upper panel). F18MOE retained its ability to trigger *POLR2H* exon 2 skipping in all three mutants carrying intronic deletions, although the effect was somewhat less pronounced in the context of the shortest intron (Fig. 4C, lower panel). To uncover if F18MOE directly binds to the predicted target sequence within *POLR2H* exon 2 (Supplementary Fig. S11), we introduced a series of mutations in the first three nucleotides of the target region with the highest complementarity to F18MOE (Fig. 4D). These mutations almost completely abrogated the negative effect of F18MOE on *POLR2H* exon 2 splicing, confirming the presence of the F18MOE binding site within *POLR2H* exon 2 (Fig. 4D). These results supported the presence of an exonic splicing enhancer (ESE) with an ISS-N1-like sequence within *POLR2H* exon 2, sequestration of which triggers exon skipping. Supporting this argument, we observed a slight increase in skipping of exon 2 in all of the mutant minigene transcripts except for G11A (Fig. 4D).

To further confirm that F18MOE sequesters positions 8 to 25 of *POLR2H* exon 2, we generated minigenes with mutations within the target sequence restoring base pairings at the 8th and 10th positions (M1 mutant), replacing the wobble base pairings with the canonical base pairings at the 16th and 20th positions (M2 mutant), and introducing perfect complementarity with all four mutations (M3 mutant) (Fig. 4E). We hypothesized that *POLR2H* exon 2 mutants with stronger base pairings with F18MOE would undergo skipping in the presence of lower concentrations of ASO. We cotransfected these mutant minigenes into HEK293 cells with increasing concentrations of F18MOE (5, 20, and 100 nM) and measured the splicing pattern of transcripts generated from minigenes. While untreated wild-type (WT) minigene transcripts showed full inclusion, we observed 20%, 68%, and 84% skipping of *POLR2H* exon 2 at 5 nM, 20 nM, and100 nM of F18MOE, respectively, with a moderate amount of intron 2 retention (Fig. 4E). Correction of both mismatches (M1 mutant) resulted in predominant skipping (63%) even at 5 nM F18MOE, while both 20 and 100 nM treatments resulted in more than 90% skipping (Fig. 4E). Converting G-T wobble base pairs to canonical A-T base pairs (M2 mutant) had an even stronger effect, while correcting all four mismatches and wobble base pairs resulted in 92% skipping at 5 nM F18MOE and complete skipping at higher concentrations.

We expanded our investigation to establish whether the off-target effect of F18MOE on splicing of *POLR2H* exon 2 is specifically due to high tolerance of MOE modifications for mismatch base pairing with the target sequence. We cotransfected HEK293 cells with *POLR2H* minigenes carrying mutations of the ISS-N1-like sequence within *POLR2H* exon 2 and increasing concentrations of F18OMe. As expected, even the highest concentration of F18OMe failed to trigger skipping of exon 2 in transcripts generated from WT *POLR2H* minigene, supporting that OMe-modified ASOs have no tolerance for mismatch base pairing with the target sequence within *POLR2H* exon 2. In contrast, all three minigene mutant transcripts that partially or fully restored base pairing with the F18OMe underwent increased skipping of exon 2 (Fig. 4F, right panel), although the effect was slightly weaker than with F18MOE. To rule out that the negative effect of F18MOE on splicing of *POLR2H* exon 2 is mediated through factors that recognize PS backbone and/or MOE modifications in the context of the annealed F18MOE with the target sequence, we treated HEK293 cells with F20PMO and WT or mutant minigenes of *POLR2H*. Considering PMOs are poorly transfected by Lipofectamine, we performed these experiments using nucleofection and high concentrations (6 µM) of F20PMO and ScrPMO. While high concentration of F20PMO caused only a slight skipping of exon 2 in WT transcripts of *POLR2H*, mutant transcripts of *POLR2H* with the increased complementarity between F20PMO underwent substantial to near complete skipping of exon 2 (Supplementary Fig. S12). These results confirmed that the off-target effect of F18MOE on splicing of *POLR2H* exon 2 is solely due to steric blocking of the exon 2 target sequence. To confirm that the effect of F18MOE on splicing of transcripts generated from *POLR2H* minigenes is not cell-type specific, we performed parallel experiments in HeLa cells and found similar results as observed with HEK293 cells (Supplementary Fig. S13).

### ISS-N1-like ESEs retain the off-target effects of F18MOE in a heterologous context

**Figure 5.**
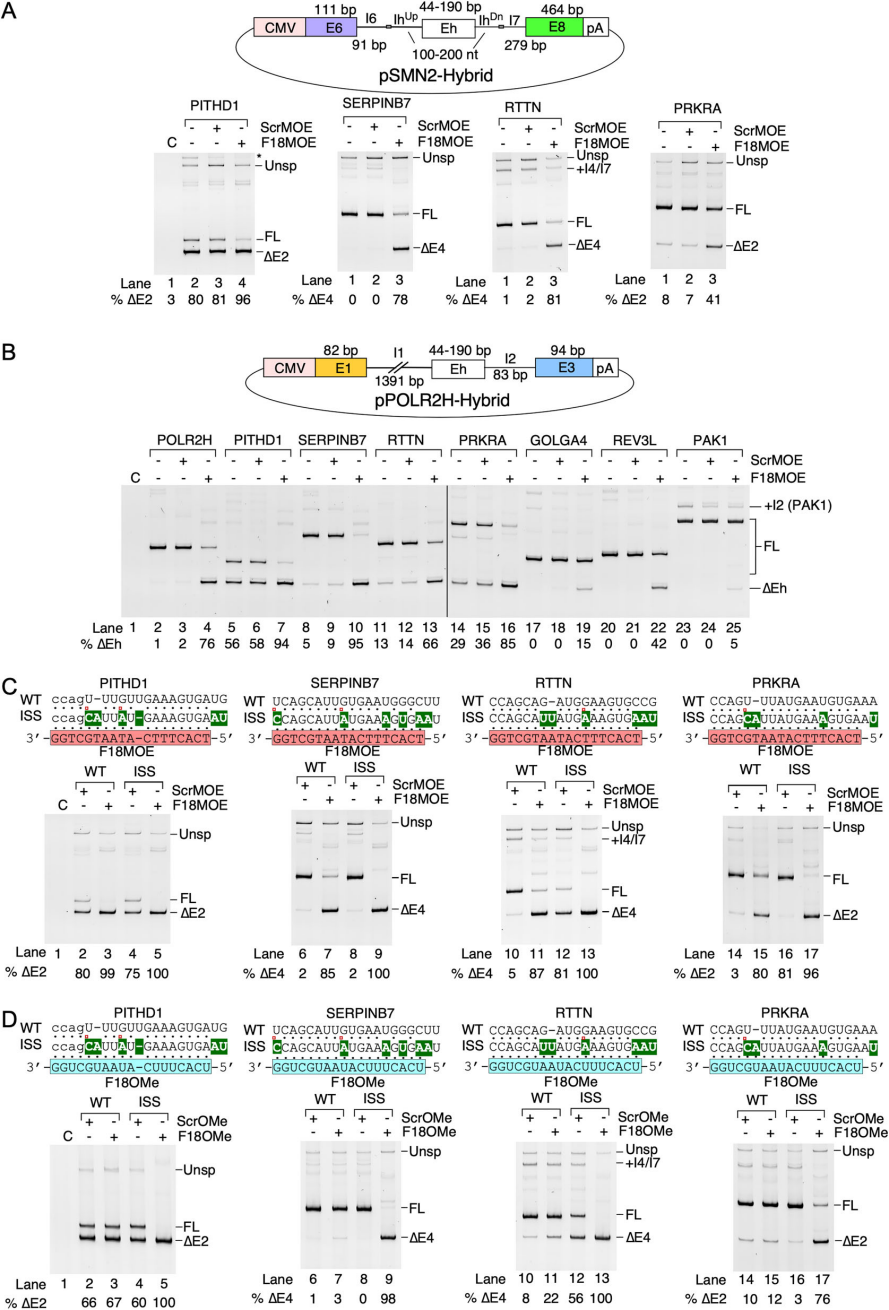
Sequence-dependent skipping of multiple exons is triggered by F18MOE through direct annealing. (**A**) Upper panel: Overview of pSMN2-Hybrid minigenes. Coloring and labeling are the same as in Fig. 4A. Lower panel: Splicing pattern of transcripts generated from hybrid minigenes cotransfected with 100 nM of the indicated ASO. Labeling is similar to Fig. 1C. Abbreviations: Eh: hybrid exon, Ih^Up^: upstream hybrid intron, Ih^Dn^: downstream hybrid intron, Unsp – unspliced product, * – nonspecific PCR product, +I – intron retained products. (**B**) Upper panel: overview of pPOLR2H-Hybrid minigenes. Lower panel: Splicing pattern of transcripts generated from hybrid minigenes cotransfected with 100 nM of the indicated ASO. (**C**) Upper panels: Predicted annealing location of F18MOE in various target exons (WT) and mutated sequences (ISS) designed to strengthen ASO annealing. Coloring and labeling are the same as Fig. 4C. Lower panels: Splicing pattern of transcripts generated from mutated hybrid minigenes cotransfected with 100 nM of the indicated ASO. (**D**) Splicing pattern of transcripts generated from mutant minigenes in the presence of F18OMe. Coloring and labeling are the same as in (C).

To determine whether F18MOE directly targets potential ESEs in other off-target genes, we generated seven hybrid minigenes using the *SMN2* minigene as a backbone. Hybrid minigenes were created by replacing *SMN2* exon 7 and its flanking intronic sequences with F18MOE-sensitive off-target exons and their flanking intronic sequences (Fig. 5A). We transfected these hybrid minigenes into HEK293 cells with or without F18MOE or ScrMOE and examined their splicing patterns (Fig. 5A and Supplementary Fig. S14A). Transcripts generated from four (*PITHD1, SERPINB7, RTTN*, and *PRKRA*) out of seven minigenes exhibited a robust increase in skipping triggered by F18MOE (Fig. 5A). Transcripts generated from *GOLGA4, REV3L*, and *PAK1* minigenes showed slight increase in exon skipping, increased intron retention, and no effect in the presence of F18MOE, respectively (Supplementary Fig. S14A). Results were consistent with splicing pattern of the endogenous exons in HEK293 cells, except for *SERPINB7*, which was not expressed (Supplementary Fig. S14B). Interestingly, *PAK1* exon 2 was also barely affected by F18MOE in the endogenous context, showing differential regulation of this exon in HEK293 cells compared to GM03813 SMA patient fibroblasts (Fig. 3B and Supplementary Fig. S14B).

To confirm that the effect of F18MOE on the splicing of off-target exons was associated with ESEs, we generated a different set of hybrid minigenes using *POLR2H* minigene as a backbone. We created seven hybrid minigenes by replacing exon 2 of *POLR2H* with other F18MOE-sensitive exons of interest while fully retaining the flanking intronic sequences of *POLR2H* minigene (Fig. 5B). We transfected the hybrid minigenes into HEK293 cells with or without F18MOE or ScrMOE and examined the splicing patterns of the resulting transcripts. Transcripts generated from all seven hybrid minigenes exhibited at least some increased skipping in the presence of F18MOE, although *GOLGA4* exon 4 and *PAK1* exon 2 were only weakly affected (Fig. 5B). Next, we examined the effect of mutations within the exons, which introduced perfect complementarity to F18MOE. We generated these mutants in the context of the *SMN2* hybrid minigenes. We transfected mutated hybrid minigenes into HEK293 cells with or without F18MOE or ScrMOE and examined their splicing patterns. Transcripts generated from all mutated hybrid minigenes showed greater skipping of exons (or intron retention in the case of REV3L) except for *PAK1*, which showed only minute skipping of exon 2 (Fig. 5C and Supplementary Fig. S14C). These results confirmed that F18MOE-induced skipping of the off-target exons is the consequence of sequestration of an ESE that resembles ISS-N1 in sequence.

To determine whether F18MOE-induced skipping of exons was due to higher tolerance for mismatches of MOE chemistry combined with the steric blocking of an ESE, we transfected HEK cells with WT or the mutated hybrid minigenes with or without F18Ome or ScrOMe and examined their splicing patterns. In almost every case, F18OMe had little or no effect on splicing of transcripts generated from the wild-type minigene (Fig. 5D and Supplementary Fig. S14D), confirming that OMe chemistry has poor or no tolerance for mismatches with the target sequence within the F18MOE-sensitve exons we examined. The only exception was *RTTN* exon 4, which showed a slight increase in F18OMe-induced skipping. In contrast, F18OMe triggered significant skipping in transcripts generated from all mutant minigenes encompassing perfect complementarity with the exception of *PAK1* exon 2 (Fig. 5D and Supplementary Fig. S14D). These results further confirmed the presence of ESEs resembling ISS-N1 in different exonic contexts and underscored that the steric blocking of these ESEs could be achieved with ASOs enforcing full complementarity regardless of chemistry, while MOE-specific effects tend to stem from a tolerance for mismatch base pairings.

### F18MOE-responsive ESEs are portable in different contexts

**Figure 6.**
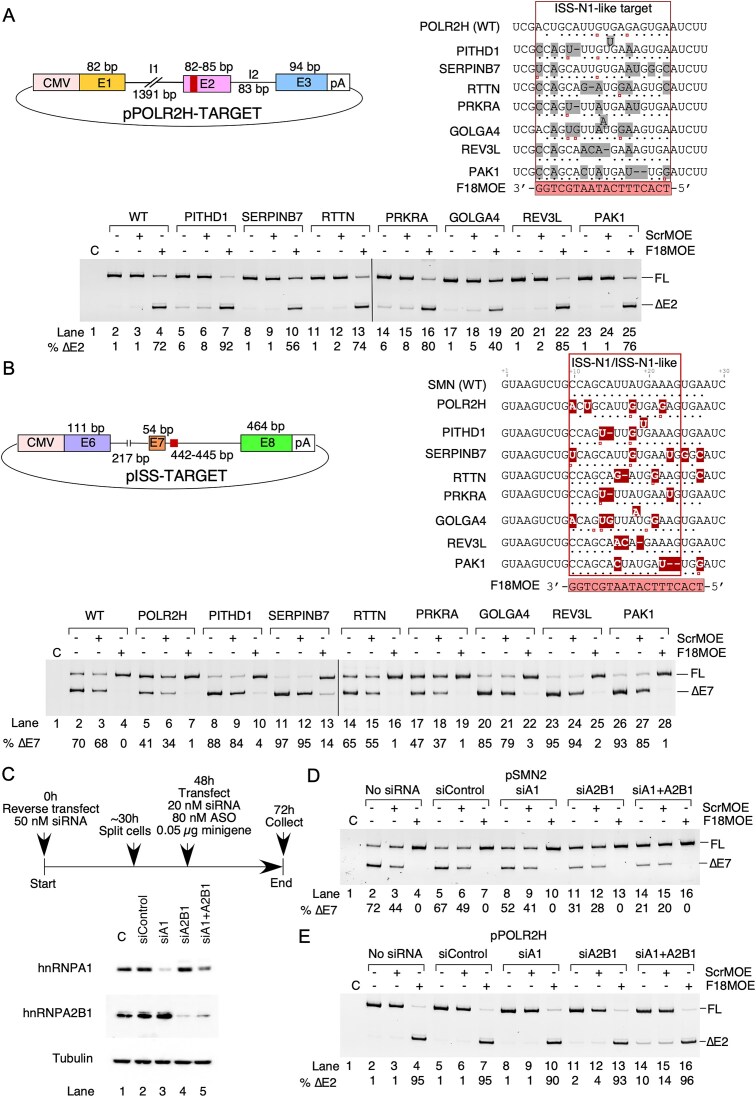
*Cis*-elements targeted by F18MOE are exchangeable and context-dependent. (**A**) Upper left panel: Overview of pPOLR2H-TARGET minigenes. Coloring and labeling are the same as Fig. 4A, except a red box in exon 2 indicates location of sequences replaced with other F18MOE-responsive ESEs. Upper right panel: predicted annealing of F18MOE to WT *POLR2H* sequence or varied target sites (boxed in red) inserted into the context of *POLR2H* exon 2 (unboxed sequences). Black dots indicate canonical base pairs with F18MOE, open red circles indicate G-U/G-T “wobble” base pairs. Lower panel: Splicing pattern of transcripts generated from pPOLR2H-TARGET minigenes cotransfected with 100 nM of the indicated ASOs. (**B**) Upper left panel: Overview of the pISS-TARGET minigenes. Coloring and labeling are the same as in (A). Upper right panel: predicted annealing of F18MOE to ISS-N1 or other F18MOE-responsive ESEs mentioned in (A). Mutated bases relative to ISS-N1 are highlighted in red. Lower panel: Splicing pattern of transcripts generated from pISS-TARGET minigenes cotransfected with 100 nM of the indicated ASOs. (**C**) Upper panel: siRNA transfection scheme to knock down hnRNPA1 and hnRNPA2B1. Lower panel: western blot to verify efficient knockdown of the target proteins. siRNA treatments are indicated at the top, specific proteins probed by antibodies are indicated at the left. (**D**) Splicing pattern of transcripts generated from pSMN2 minigene cotransfected with 100 nM of the indicated ASOs after knockdown of hnRNPA1 and/or hnRNPA2B1. (**E**) Splicing pattern of transcripts generated from pPOLR2H minigene cotransfected with 100 nM of the indicated ASOs after knockdown of hnRNPA1 and/or hnRNPA2B1.

We next examined whether the F18MOE-responsive ESEs with varied sequences and diverse exonic contexts are portable. We generated mutant minigenes of *POLR2H* in which the F18MOE-responsive ESE within exon 2 was replaced with one of the other seven F18MOE-responsive ESEs (Fig. 6A). We transfected these mutant minigenes into HEK293 cells with or without F18MOE or ScrMOE and examined their splicing patterns. Transcripts generated from all seven mutants showed F18MOE-induced skipping (40%–92%), whereas the control ASO had no effect (Fig. 6A). The effects of F18MOE on exon skipping correlated well with the number of canonical base pairs formed between the ASO and its targeted ESE. In particular, the transcript generated from the minigene carrying the *SERPINB7* target ESE, which formed 13 canonical base pairs with F18MOE, exhibited a relatively low amount of skipping (Fig. 6A, lanes 10 and 19, and Supplementary Table S5), while the transcripts of minigenes carrying ESEs from *PITHD1, REV3L*, and *PRKRA*, which formed 15 canonical base pairs, responded strongly to ASO (Fig. 6A, lanes 7, 16, and 22, and Supplementary Table S5). The transcript generated the minigene carrying the *GOLGA4* ESE was a notable exception, as it is predicted to form 14 canonical base pairs with F18MOE yet only underwent 40% skipping in the presence of F18MOE.

We also tested the effects of the F18MOE-responsive ESEs in the intronic context to determine if ESEs could behave like intronic splicing silencers (ISSs), similar to that of ISS-N1. We generated mutant minigenes of *SMN2* in which ISS-N1 within intron 7 was replaced with one of the eight F18MOE-responsive ESEs (Fig. 6B). Each mutation changed at least three bases within the ISS-N1 sequence. However, the inhibitory action of ISS-N1 on *SMN2* exon 7 splicing was retained, as all of the mutant minigene transcripts still showed *SMN2* exon 7 skipping in the absence of an ISS-N1-targeting ASO. F18MOE completely or nearly completely restored *SMN2* exon 7 splicing in all mutant minigene transcripts, even in cases where complementarity was poor (Fig. 6B). These results confirmed that ESEs with diverse sequences have potential to become ISSs and location of such sequences is the prime determinant of their function.

It has been proposed that the inhibitory effect of ISS-N1 is associated at least in part to binding by hnRNPA1 and/or hnRNPA2B1 [52]. It has also been shown that hnRNPA1/A2B1 could serve as both a negative and positive regulator of splicing depending upon the context [53]. To determine if the stimulatory effect of ISS-N1-like ESEs is associated with hnRNPA1/A2B1, we examined the splicing pattern of transcripts generated from *SMN2* and *POLR2H* minigenes in HEK293 cells depleted of hnRNPA1 or hnRNPA2B1, or both by siRNAs (Fig. 6C–E). We confirmed efficient knockdown of both proteins by western blot, although double knockdown was slightly less effective than single knockdown of each protein (Fig. 6C). Supporting the previous findings, depletion of hnRNPA1/A2B1 had a stimulatory effect on inclusion of exon 7 in transcripts generated from *SMN2* minigene (Fig. 6D). A similar result was obtained in transcripts generated from endogenous *SMN2* (Supplementary Fig. S15A). However, knockdown of hnRNPA1 and/or hnRNPA2B1 only produced a slight negative effect on splicing of exon 2 in transcripts generated from *POLR2H* minigene (Fig. 6E). A similar result was obtained in transcripts generated from endogenous *POLR2H* (Supplementary Fig. S15B). We next evaluated the effects of hnRNPA1/A2B1 knockdown on splicing of additional F18MOE-responsive exons in transcripts generated from endogenous genes. Simultaneous knockdown of hnRNPA1 and hnRNPA2B1 mildly increased skipping of *REV3L* exon 9 and *PRKRA* exon 2, while in contrast it was slightly stimulatory for *GOLGA4* exon 4 splicing (Supplementary Fig. S15C). There was no observable effect on splicing of other exons that we examined (Supplementary Fig. S15C). Overall, while findings supported mild stimulatory effect of hnRNPA1/A2B1 on splicing of a subset of exons harboring ISS-N1-like ESEs, additional factors are likely to be associated with these ESEs.

In order to identify other RNA binding proteins potentially interacting with ISS-N1-like ESEs, we analyzed the sequences of ISS-N1 and eight nusinersen-responsive sequences along with five upstream and downstream nucleotides using DeepCLIP (Supplementary Table S6) [38]. We did not observe any proteins with consistently high scores among all target sequences, suggesting that there may not be a single shared RNA-binding protein interacting with all nusinersen-responsive *cis* elements. The highest-scoring potential interactions (scores > 0.9) were between ALKBH5 and *RTTN*, ALKBH5 and *PAK1*, G3BP1 and *RTTN*, SRSF1 and *RTTN*, TARDBP and *PRKRA*, and TRA2A and *RTTN*. We noted that the *RTTN* sequence scored consistently highly among a wide range of RNA-binding proteins, which may indicate a systemic bias in DeepCLIP models or CLIP methodologies causing certain sequences to have inflated scores.

### Short MOE ASOs require full complementarity with the target to retain off-target effects

**Figure 7.**
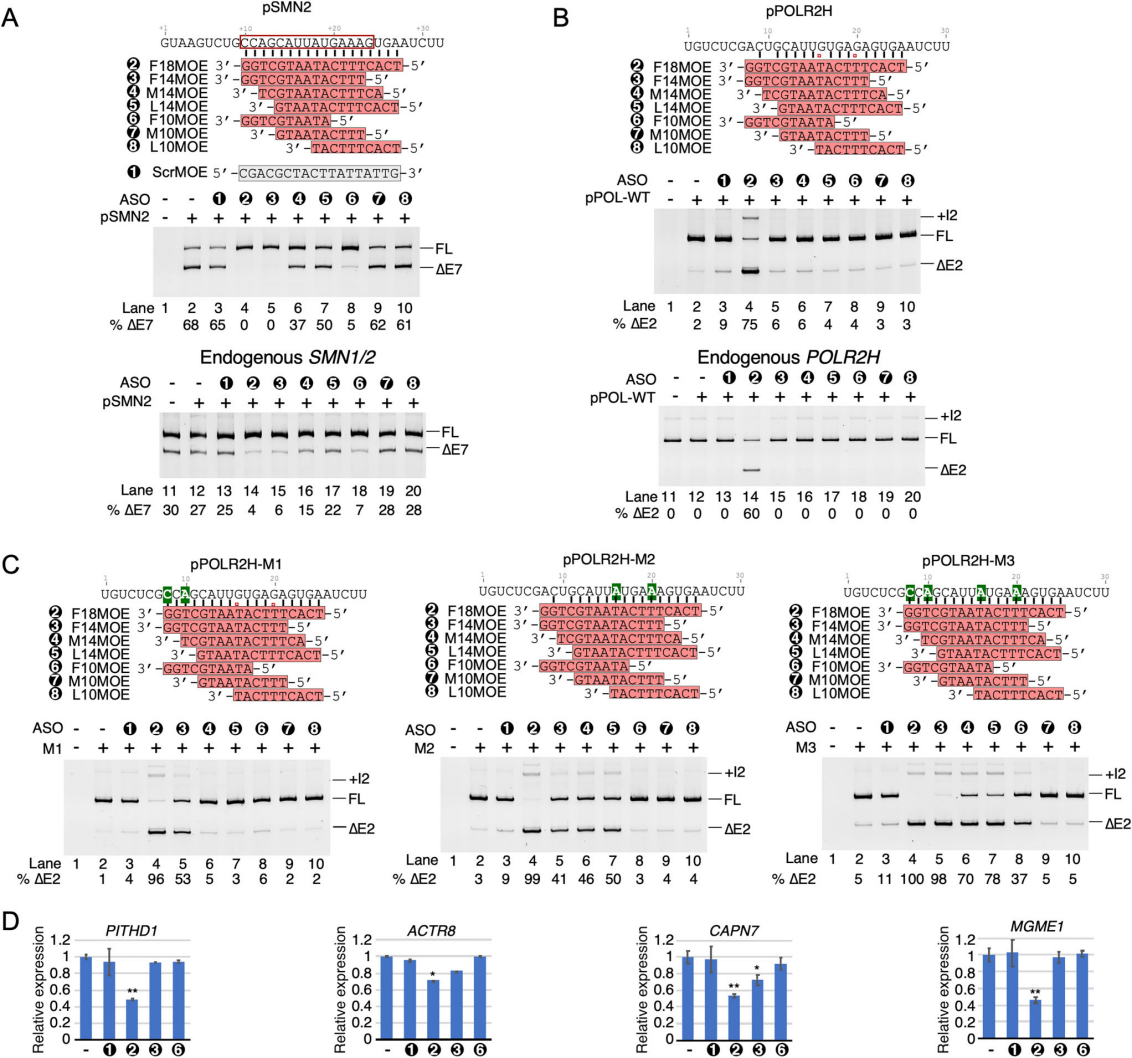
Short ASOs targeting ISS-N1 have reduced off-target effects. (**A**) Upper panel: Overview of F18MOE and shorter ASOs annealing to ISS-N1 within *SMN2* intron 7. ASOs are numbered, which is used throughout the figure. Middle panel: Splicing pattern of transcripts generated from pSMN2 in HEK293 cells cotransfected with the indicated ASOs. Treatments are indicated at the top of the gel. Labeling is the same as Fig. 1C. Lower panel: Splicing pattern of endogenous *SMN1/2* in HEK293 cells transfected with the indicated ASOs. (**B**) Upper panel: Overview of F18MOE and shorter ASO variants annealing to *POLR2H* exon 2 in the pPOLR2H minigene. Labeling and coloring is the same as in Fig. 4C. Middle panel: Splicing pattern of transcripts generated from pPOLR2H minigene in HEK293 cells cotransfected with the indicated ASOs. Lower panel: Splicing pattern of endogenous *POLR2H* in HEK293 cells transfected with the indicated ASOs. Labeling is the same as in panel (A). (**C**) Splicing pattern of transcripts generated from mutated pPOLR2H minigenes with improved annealing to F18MOE in the presence of shorter ASO variants. Mutated bases are highlighted in green. (**D**) Relative expression of four genes upon treatment with 18-mer, 14-mer, or 10-mer ISS-N1-targeting ASOs, as measured by qPCR. *P < 0.05, **P < 0.01. Error bars represent SEM.

We employed short ASOs encompassing MOE modifications to uncover if the off-target effect of F18MOE was due to sequestration of the structurally accessible smaller motifs within the F18MOE-responsive ESE. We also wanted to test if short ASOs encompassing MOE modifications tolerate mismatch base pairing while eliciting the off-target effects. We performed these experiments in HEK293 cells focusing on *POLR2H* exon 2 and monitored the effects of short MOE ASOs in the context of both *POLR2H* minigene and endogenous *POLR2H*. As a control, we used *SMN2* to confirm the on-target (ISS-N1-specific) effects of short MOE ASOs. Of note, a previous study employing a short OMe ASO sequestering the 8-nucleotide-long GC-rich motif overlapping the first five residues of ISS-N1 has been shown to promote *SMN2* exon 7 inclusion [26]. It has also been demonstrated that the sequestration of the first 14 residues of ISS-N1 by a 14mer ASO encompassing OMe modifications promotes inclusion of exon *SMN2* exon 7 by disrupting a RNA structure formed by a long-distance interaction [12, 54]. Consistent with these findings, a 14mer MOE ASO sequestering the first 14 residues of ISS-N1 fully restored *SMN2* exon 7 inclusion and the other 14mer MOE ASOs targeting middle and downstream motifs of ISS-N1 had intermediate and weak stimulatory effects on *SMN2* exon 7 splicing, respectively (Fig. 7A) [54]. Also consistent with the previous findings, a 10mer MOE sequestering the first 10 residues of ISS-N1 fully restored *SMN2* exon 7 inclusion, while the 10mer MOE ASOs targeting middle and downstream sequences had no effect on *SMN2* exon 7 splicing (Fig. 7A) [26]. In contrast, none of the short MOE ASOs retained the off-target effects on splicing of *POLR2H* exon 2, supporting that short ASOs are unable to tolerate mismatch base pairing with the F18MOE-responsive ESE (Fig. 7B). Notably, L14MOE that sequestered the 3′-end of the F18MOE-responsive ESE failed to elicit any off-target effect despite the fact that this ASO possessed the potential to form a continuous 14-base pair RNA–RNA duplex, albeit encompassing wobble base pairs (Fig. 7B). These results indicated that the sequestration of small motifs located toward the 5′-end of the F18MOE-responsive ESE could be the major driver for the off-target effect of F18MOE or that wobble base pairs are less well tolerated than previously assumed. We observed similar results in HeLa cells, supporting that the effect of short MOE ASOs is not specific to any particular cell type (Supplementary Fig. S16).

We performed the next set of experiments using the three mutant *POLR2H* minigenes with increased complementarity to F18MOE (Fig. 4E). Except for F14MOE that partially retained the negative effect on splicing of exon 2 in transcripts generated from M1, none of the other short ASOs produced any inhibitory effect on splicing of M1 transcripts (Fig. 7C). Results suggested that small motifs toward the 5′-end of the F18MOE-responsive ESE are critical for the off-target effect. The lack of the strong negative effect of F14MOE could be attributed to the presence of the wobble base pairing toward the 3′-end of the F18MOE-responsive ESE. While all three 14mer ASOs exerted a similar level of partial negative effects on splicing of M2 transcripts, none of the 10mer ASOs had an effect on splicing of M2 transcripts. Lack of a strong negative effect by any of the 14mer ASOs on splicing of M2 transcripts could be attributed to the fact that the 5′-end of the F18MOE-responsive ESE was not sequestered. Four ASOs, namely F14MOE, M14MOE, L14MOE, and F10MOE exerted a negative effect on splicing of transcripts generated from M3, whereas M10MOE and L10MOE had no appreciable effect (Fig. 7C). These results supported that the simultaneous sequestration of at least two motifs, the CAUUAU motif spanning from 5th to 10th position of F18MOE-responsive ESE and an upstream or a downstream motif, is critical for the inhibitory effects of the short MOE ASOs targeting F18MOE-responsive ESE.

We also examined the effect of short ASOs encompassing MOE modifications on transcription of four genes (*PITHD1, ACTR8, CAPN7*, and *MGME1*) that were downregulated by F18MOE. In particular, we used F14MOE and F10MOE, representing 14mer and 10mer ASOs, respectively, due to their ability to exert a negative effect on *POLR2H* exon 2 splicing in M3 transcripts (Fig. 7C). We performed this experiment by transfecting HEK293 cells with 100 nM of ASOs. As expected, F18MOE reduced expression of all four genes (Fig. 7D). Out of four genes examined, only *CAPN7* was slightly downregulated by F14MOE, whereas F10MOE had no effect on expression of any of the genes examined (Fig. 7D). These results supported that the off-target effect of F18MOE on transcription was at least in part due to the large size of this ASO.

### MOE modifications at specific positions of ASOs are critical determinants of off-target effects

**Figure 8.**
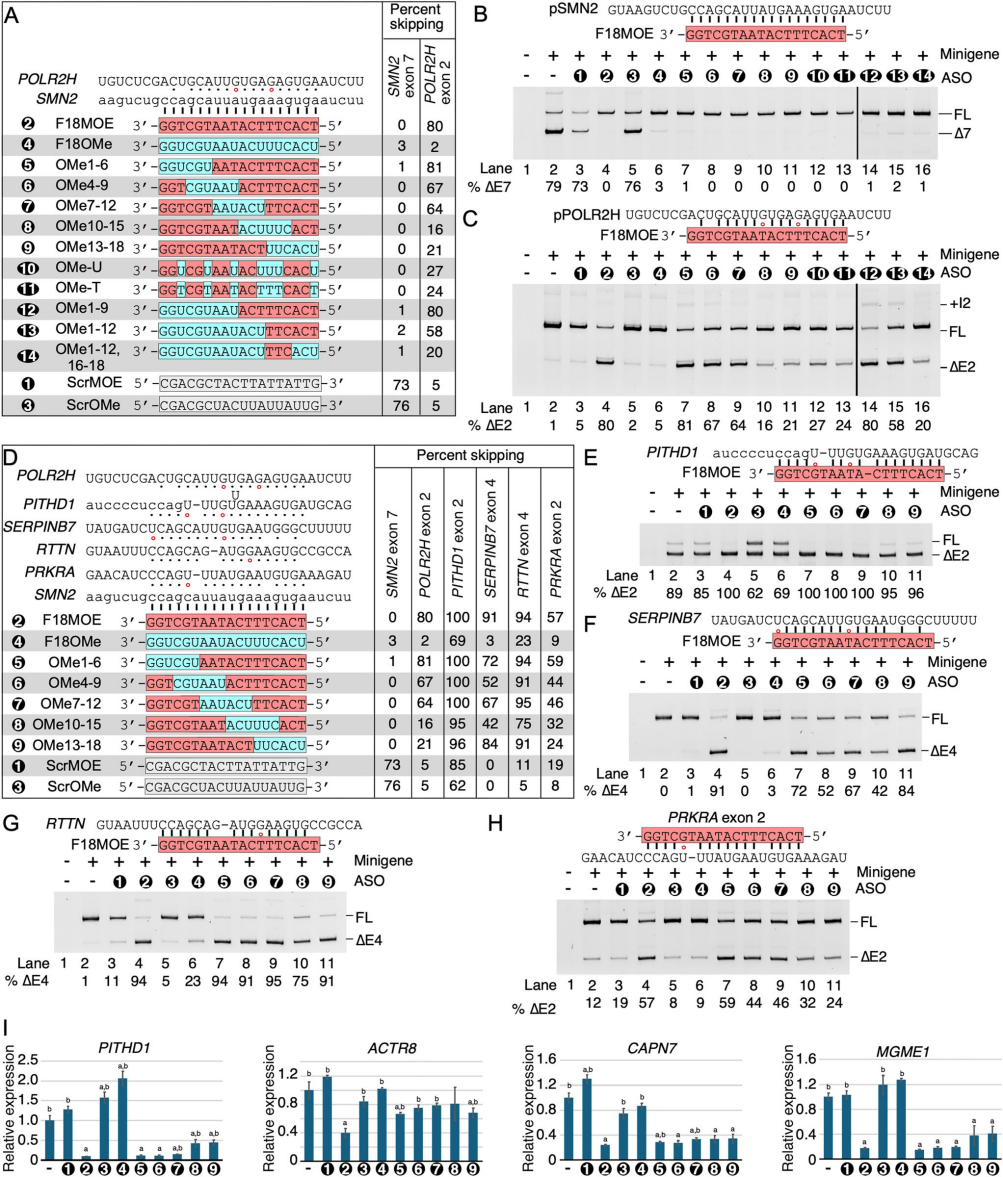
ASOs with mixed chemistry have reduced off-target effects. (**A**) Sequences of mixed chemistry ASOs and their annealing locations are shown on the left. MOE bases are highlighted in salmon while OMe bases are highlighted in blue. Canonical base pairs are shown as black lines or dots while G:U/G:T wobble base pairs are shown as open red circles. The percentage skipping of *SMN2* exon 7 and *POLR2H* exon 2 upon treatment with the indicated ASOs is shown on the right. (**B**) Splicing pattern of transcripts derived from pSMN2 minigene cotransfected with 100 nM of the indicated ASOs. Labeling is the same as Fig. 1C. (**C**) Splicing pattern of transcripts generated from pPOLR2H minigene cotransfected with the indicated ASOs. (**D**) Sequences of mixed chemistry ASOs and annealing locations are shown on the left. The percentage skipping of the exons in transcripts generated from the indicated minigenes after treatment are indicated on the right. (**E–H**) Splicing pattern of transcripts generated from the *PITHD1* (panel E), *SERPINB7* (panel F), *RTTN* (panel G), and *PRKRA* (panel H) hybrid minigenes cotransfected with the indicated mixed-chemistry ASOs. (**I**) Relative expression of four genes upon treatment with the indicated mixed-chemistry ASOs, as measured by qPCR. “a” indicates a statistically significant (*P* < 0.05) change from untreated cells, “b” indicates a statistically significant change from F18MOE-treated cells.

To determine if MOE modifications at specific positions of ASOs are critical determinants of off-target effects, we employed a library of 18mer ISS-N1-targeting ASOs with mixed chemistries in which MOE modifications were replaced with OMe modifications at different positions while retaining the PS backbone (Fig. 8A). Since OMe and MOE modified ASOs used U and T residues, respectively, we also compared the effect of OMe-modified Us and Ts in ISS-N1-targeting ASOs. We cotransfected HEK293 cells with 100 nM of a given ASO and *SMN2* minigene or *POLR2H* minigene. As expected, all ISS-N1-targeting ASOs encompassing mixed modifications efficiently restored *SMN2* exon 7 inclusion (Fig. 8B). However, we observed a varied response on splicing of *POLR2H* exon 2. For instance, OMe1-6 that had MOE-to-OMe replacement at the first six positions from the 3′-end of the ASO fully retained the off-target effect on splicing of *POLR2H* exon 2. OMe4-9 and OMe7-13 that had MOE-to-OMe replacement from 4th to 9th and 7th to 13th positions from the 3′-end of the ASO, respectively, showed reduced inhibitory effect on splicing of *POLR2H* exon 2. In case of OMe10-15 and OMe13-18 that had MOE-to-OMe replacement from 10th to 15th and 13th to 18th positions from the 3′-end of the ASO, respectively, substantially diminished the inhibitory effect on splicing of *POLR2H* exon 2 (Fig. 8C). These results supported that MOE modifications toward the 5′-half of the ISS-N1-targeting ASO are critical for the off-target effects on splicing of *POLR2H* exon 2. Replacement of T residues in F18MOE with OMe-U or OMe-T had similar reductions in the off-target effect, supporting that the effect is primarily due to the presence of MOE modification, and not due to T residues (Fig. 8C).

To determine the minimal number of MOE-modified bases required for eliciting the off-target effect on *POLR2H* exon 2 splicing, we used three ISS-N1-targeting ASOs, namely OMe1-9, OMe1-12, and OMe1-12/16-18, encompassing an increasing number of OMe modifications while reducing the number of MOE modifications. We observed a very small decrease in the off-target effect on *POLR2H* exon 2 splicing with OMe1-9 that had nine residues with MOE modifications at the 5′-end of the ASO (Fig. 8C). OMe1-12 that had six residues with MOE modifications at the 5′-end of ASO showed a further decrease in the off-target effect and yet >50% transcripts of *POLR2H* underwent exon 2 skipping (Fig. 8C). However, we captured a drastic decrease in off-target skipping of *POLR2H* exon 2 by OMe1-12/16-18 that encompassed only three MOE modifications spanning from 13th to 15th positions from the 3′-end of ASO (Fig. 8C). Therefore, we conclude that at least six uninterrupted MOE-modified nucleotides are required to trigger a robust off-target effect on splicing of *POLR2H* exon 2.

We compared the effect of ISS-N1-targeting ASOs encompassing mixed modifications on splicing of five F18MOE-responsive exons, namely *POLR2H* exon 2, *PITHD1* exon 2, *SERPINB7* exon 4, *RTTN* exon 4, and *PRKRA* exon 2 (Fig. 8D–H). We performed this experiment employing five ASOs carrying MOE-to-OMe replacements at six continuous positions in different regions of the ISS-N1-targeting ASO. All ISS-N1-targeting ASOs encompassing mixed modifications promoted skipping of *PITHD1* exon 2 except OMe10-15 and OMe13-18 which caused a slightly reduced skipping (Fig. 8E). *SERPINB7* exon 4 exhibited the greatest reduction in skipping by OMe4-9 and OMe10-15 (Fig. 8F). In the case of *RTTN* exon 4, all ASOs encompassing mixed chemistries, except for OMe10-15 which showed a slight decrease in off-target effect, fully retained the off-target effect on splicing (Fig. 8G). *PRKRA* exon 2 showed a similar pattern to that of *POLR2H* exon 2, with the MOE modifications at the 3′-end of the ASO being critical for the off-target effect (Fig. 8H). Overall, the requirement for MOE modifications at different positions within the ISS-N1-targeting ASO varied for eliciting the off-target effect on splicing of different exons, although in each case OMe10-15 produced one of the greatest reductions in off-target effects compared to F18MOE, suggesting these positions are generally important for annealing.

We examined whether ISS-N1-targeting ASOs encompassing mixed modifications have an effect on the expression of transcripts that were downregulated by F18MOE. Here again, we employed five ASOs encompassing MOE-to-OMe replacements at six continuous positions in different regions of the ISS-N1-targeting ASO. We performed this experiment in HeLa cells and monitored the levels of *PITHD1, ACTR8, CAPN1*, and *MGME1* transcripts that were downregulated by F18MOE but not by other ASOs. Interestingly, all ASOs encompassing mixed modifications retained the inhibitory effect on expression of *PITHD1, CAPN1*, and *MGME1*, supporting that even sparse MOE modifications within ASO is also capable of triggering off-target effects on expression of a subset of genes (Fig. 8I). In contrast, all ASOs encompassing mixed modifications had reduced inhibitory effect on expression of *ACTR8*, supporting that the continuity of MOE modifications within ASO is required to elicit off-target effect on expression of another subset of genes.

## Discussion

The discovery of ISS-N1 in 2006 heralded the ASO-mediated splicing correction for the treatment of SMA, the most frequent genetic cause of infant mortality. Nusinersen, an ISS-N1-targeting ASO, became the first drug for the treatment of SMA in 2016. While the mechanism by which nusinersen promotes *SMN2* exon 7 inclusion has been extensively investigated [55], limited attention has been paid toward its off-target effects. Here we report transcriptome-wide alterations caused by F18MOE that possesses identical sequence and chemical composition to that of nusinersen. While the majority of genes impacted by F18MOE were also affected by ScrMOE, a small proportion of genes were impacted by all four ASOs encompassing MOE or OMe modifications. These results supported that the hybridization independent effect of F18MOE played a major role in changes in the transcriptome. Nonetheless, F18MOE uniquely upregulated and downregulated 16 and 86 genes, respectively, supporting that the sequence-specific effects also played an important role in F18MOE-associated perturbation of the transcriptome. Consistent with a recent study implicating the role of an ISS-N1-targeting ASO encompassing MOE modifications in transcription of *SMN1/2* genes through interaction with the chromatin structure [51], several genes upregulated by F18MOE were clustered in the chr11q22.2 region. Additional mechanisms including secondary effects may account for the broad perturbation of the transcriptome. Among cellular processes most strongly associated with transcripts affected by F18MOE were cell cycle, cell growth, cell signaling, and maintenance of cytoskeleton, chromosomes and organelles.

We independently evaluated by qPCR the aberrant expression of 38 genes that were found to be impacted by F18MOE in our RNA-Seq analysis (Fig. 2). Among genes that we validated to be significantly downregulated by 6 μM of F18MOE in a sequence-dependent manner were *PITHD1, WDR70, ATP5ME, COPG2, MAGED2, ACTR8, CAPN7, MGME1*, and *TMCO3*. Although we evaluated a similar number of genes expected to be upregulated upon F18MOE treatment in a sequence-dependent manner, significant upregulation was captured only in the case of *STAG1* and *JNF561* and none of these genes were impacted by lower concentrations of F18MOE. These results suggested that the sequence-dependent downregulation but not upregulation is the defining feature of F18MOE. Confirming this, when we treated HeLa cells with F18MOE and commercially available nusinersen by lipofectamine-mediated transfection, we only observed significant changes in expression among genes that were downregulated by F18MOE/nusinersen (Supplementary Fig. S4). Among genes that we validated to be significantly upregulated by 6 μM of F18MOE in a sequence-independent manner were *PRSS3, IRAK1, PPAT, TMEM97, MMP16*, and *MICAL2*. For *TMEM97, MMP16*, and *MICAL2*, we captured their upregulation even at 1.5 μM of F18MOE. These results supported that any ASO encompassing PS backbone and MOE modifications is capable of upregulating a subset of genes likely by specifically associating with the gene body and/or transcripts and/or factors associated with transcription in a sequence-independent manner. Several of the gene expression changes recorded in SMA patient fibroblasts were also captured in HeLa, HEK293, and neuronal SH-SY5Y cells, suggesting a body-wide perturbation of the transcriptome at high concentrations of F18MOE. The broad spectrum of genes confirmed to be aberrantly expressed by F18MOE are predicted to adversely affect critical cellular functions, including DNA replication, DNA repair, transcription, pre-mRNA splicing, mRNA transport, mRNA turnover, translation, signal transduction, macromolecular trafficking, stress-granule formation, cytoskeletal dynamics, and protein turnover (Supplementary Fig. S3 and Supplementary Table S3).

We next analyzed the effects of ISS-N1-targeting ASOs on alternative splicing and found moderate perturbation of exon skipping and inclusion by all three chemistries (Fig. 3). Using semiquantitative PCR, we validated 8 randomly selected skipping events triggered by F18MOE, including skipping of *POLR2H* exon 2, *PITHD1* exon 2, *SERPINB7* exon 4, *RTTN* exon 4, *REV3L* exon 9, *PRKRA* exon 2, *GOLGA4* exon 4, and *PAK1* exon 2 (Fig. 3B). In all cases, the effect was captured even at the lowest F18MOE concentration, whereas other ASOs did not have any impact on splicing of the above exons even at the highest concentration used. Sizes of the skipped exons and their flanking intronic sequences greatly varied, suggesting that skipping was solely driven by specific sequences responsive to F18MOE. We could validate only 2 of the 7 randomly selected F18MOE-induced inclusion events, particularly inclusion of *ZNF561* exon 4 and *GFM1* exon 6, suggesting the limitations of semiquantitative PCR in capturing mild effects (Fig. 3C and Supplementary Fig. S9). Similarly, we were unable to validate any of the F20PMO-induced skipping or inclusion events, supporting a mild to negligible effect of F20PMO on splicing. Overall, findings underscored a broad distribution of F18MOE-responsive sequences, with the strongest effects and highest validation rates for exon skipping events.

To further investigate the mechanism of the off-target effects of F18MOE on splicing, we employed *POLR2H* as a model and generated a minigene encompassing exons 1–3 of *POLR2H*. Supporting the suitability of *POLR2H* for our study, the splicing pattern of transcripts generated from *POLR2H* minigene was similar to that of the transcripts generated from endogenous *POLR2H* in both HEK293 and HeLa cells (Fig. 4 and Supplementary Fig. S13). Using deletions and point mutations, we confirmed the presence of F18MOE-responsive sequence within *POLR2H* exon 2. Interestingly, F18OMe and F20PMO were also able to trigger skipping of *POLR2H* exon 2 when the target sequence was mutated to restore base pairing with each ASO. Findings ruled out that the negative effect of F18MOE on splicing of *POLR2H* exon 2 was due to inhibitory factors deposited on RNA:RNA duplex formed between F18MOE and the target site. Instead, the off-target effect of F18MOE on *POLR2H* exon 2 splicing appears to be due to MOE-specific tolerance for two mismatch base pairings at the 3′-end and two wobble base pairings in the middle of ASO (Fig. 4, and Supplementary Figs S12 and S13). Employment of hybrid minigenes confirmed the exonic locations of F18MOE-responsive elements for all exons that underwent F18MOE-induced skipping (Fig. 5). However, locations of F18MOE-responsive elements and positions of mismatch base pairing between F18MOE and the target sequence greatly varied in different exons. In many instances, F18MOE triggered off-target exon skipping despite the presence of additions and/or deletions in the target sequence, supporting a tolerance for asymmetric bulges in the RNA:RNA duplex formed between F18MOE and the target sequence. Similar to *POLR2H* exon 2, increased complementarity of F18MOE with the target sequences enhanced exon skipping for all off-target exons except for *PAK1* exon 2, while perfect complementarity also allowed F18OMe to trigger exon skipping and/or intron retention (Fig. 5 and Supplementary Fig. S14). Findings represent the first comprehensive study uncovering a broad spectrum of mismatch base pairing tolerated by a therapeutic oligonucleotide encompassing MOE modifications at every position.

All ISS-N1-like sequences uncovered in this study retained their respective location-specific characteristics in varied contexts. For instance, we were able to replace F18MOE-responsive target sequence of *POLR2H* exon 2 with other validated F18MOE-responsive target sequences and the inhibitory effect of F18MOE on *POLR2H* exon 2 splicing was retained. These results confirmed the role of a rather diverse set of ISS-N1-like sequences as ESEs. On the contrary, replacement of ISS-N1 within intron 7 of *SMN2* with F18MOE-responsive exonic target sequences retained the stimulatory effect of F18MOE on splicing of *SMN2* exon 7. These results confirmed the role of diverse ISS-N1-like sequences as intronic splicing silencers (Fig. 6). Interaction of hnRNP A1/2 proteins with ISS-N1 has been proposed as one of the potential mechanisms behind skipping of *SMN2* exon 7. However, depletion of hnRNP A1/2 had mild to no effect on splicing F18MOE-responsive exons, supporting the role of additional factors interacting with ISS-N1-like sequences encompassing ESEs.

We examined if F18MOE-responsive sequences within *POLR2H* exon 2 also respond to small ASOs encompassing PS backbone and MOE modifications. Our results confirmed that the effect of F18MOE on splicing of *POLR2H* exon 2 is dependent on large ASO size that allowed tolerance for mismatch base pairing with the target sequence. Truncation of 18mer ASO from either end to a 14mer ASO fully eliminated the inhibitory effect on splicing of *POLR2H* exon 2 (Fig. 7B). However, the same 14mer ASOs regained the inhibitory effect on *POLR2H* exon 2 splicing when full complementarity with the target sequence was restored. We observed mixed results with 10mer ASOs encompassing PS backbone and MOE modifications. For example, while a 10mer ASO with full complementarity with the 5′-end of the ISS-N1-like target within *POLR2H* exon 2 triggered skipping of *POLR2H* exon 2, another 10mer ASO with full complementarity with the 3′-end of the ISS-N1-like target had no effect. These results supported that sequestration of motif(s) toward the 5′-end of the ISS-N1-like target is critical for skipping of *POLR2H* exon 2, although it is also possible that the annealing properties of the different portions of the target sequence play a role. We also examined the effect of two small ASOs encompassing PS backbone and MOE modifications, namely 14mer F14MOE and 10mer F10MOE, on levels of transcripts that were downregulated by F18MOE (Fig. 7D). Except for *CAPN7* transcript that showed mild downregulation by F14MOE, levels of none of the transcripts were significantly affected by small ASOs. These results underscored the large size of F18MOE is responsible at least in part for triggering a broad perturbation of the transcriptome.

We employed ISS-N1-targeting ASOs with mixed modifications to determine if replacement of MOE modifications with OMe modifications at specific positions of ASO eliminate the off-target effect on *POLR2H* exon 2 splicing (Fig. 8). Our results supported that MOE modifications toward the 5′-half of the ASO is critical for triggering *POLR2H* exon 2 skipping. We also confirmed that the off-target effect of F18MOE on splicing of *POLR2H* exon 2 is not due to thymine residues present within F18MOE. Replacement of six continuous positions of MOE modifications with OMe modifications toward the 5′-half of the ASO reduced the magnitude of its off-target effect with the maximum reduction observed when MOE-to-OMe substitutions were inserted from 4th to 6th positions from the 5′-end of ASO (Fig. 8C). Considering 6th position forms a wobble base pairing with the target sequence, it is likely that the high tolerance of MOE modification for this wobble base pairing is a major contributor toward *POLR2H* exon 2 skipping. However, MOE-to-OMe substitutions at other positions also eliminated the inhibitory effect on *POLR2H* exon 2 splicing, supporting the important role of multiple interdependent MOE modifications in eliciting an off-target effect on *POLR2H* exon 2 splicing. A comparison of the off-target effect of ASOs encompassing mixed modifications on splicing of F18MOE-senstive exons supported the critical role of MOE modifications in different regions of ASO. Similar results were obtained when we examined the effect of the ASOs encompassing mixed modifications on the expression of F18MOE-sensitive genes.

Oligonucleotide annealing is generally considered to follow a two-step process: “seeding” consisting of simultaneous association of 3–7 contiguous nucleotides, followed by “zippering” of the two strands extending from the seed region [56]. Consistent with this model, none of the off-target annealing sites of F18MOE/nusinersen contain mismatched or bulged regions of more than three contiguous nucleotides (Supplementary Fig. S11), which would be considered an unsurmountable barrier for the zippering process. All of the off-target annealing sites contain at least one G-U/G-T wobble base pair. Although G-U base pairs are considered to be nearly equivalent to canonical Watson–Crick base pairs in terms of stability, they do possess unique electrostatic and structural properties [57, 58]. Surprisingly, mutating the two wobble base pairs in the *POLR2H* target sequence to canonical A-U/A-T base pairs was even more effective than correcting two mismatches to F18MOE in triggering exon 2 skipping in the presence of F18MOE (Fig. 4), suggesting that G-U/G-T wobble base pairs may not be as well tolerated in oligonucleotide annealing as previously thought. In addition, a 14-mer ASO targeting the last 14 positions of the nusinersen target sequence had no effect on *POLR2H* exon 2 skipping despite perfect complementarity aside from two wobble base pairs, while mutating the target site to restore canonical base pairing allowed the same ASO to trigger skipping (Fig. 7B and C). It is not currently known how wobble base pairs may be differentially tolerated in the context of different chemistries or whether they preferentially affect oligonucleotide seeding or zippering.

Minimizing the off-target effects of ASOs while maintaining full activity on their intended target is a complex task that requires careful examination of the sequence, chemistry, and delivery method of ASOs. Often, the very characteristics of ASOs that contribute to their effectiveness also lead to additional off-target effects. For example, the ability of phosphorothioate backbones to bind cellular and extracellular proteins is essential for ASO distribution and activity, but also contributes to their off-target effects [25, 29, 30]. Likewise, conjugating a PMO ASO to an octa-guanidine-dendrimer designed to improve tissue delivery increased its efficacy in a mouse *in vivo* study but increased retinal toxicity [59]. In the case of F18MOE/nusinersen, it appears that the extremely high affinity of MOE-modified ASOs for their target sequences is contributing to their ability to bind off-target sequences containing mismatches. Possible solutions which we explored were reduction of ASO size and use of mixed ASO chemistry, which both reduced off-target effects on alternative splicing and have been used successfully in the past (Figs 7 and 8) [20, 28]. Recently, studies have been carried out to identify mixed-chemistry ASO designs that increase target affinity [60]. However, is not currently known how these mixed chemistry designs might impact the level of off-target splicing events, as they may increase affinity equally for both on-target and off-target events.

Nusinersen has been a successful drug for preventing death of children affected by SMA, yet concerns of adverse effects, including pyrexia, vomiting, constipation, and elevated markers of kidney dysfunction have been reported [19, 61, 62]. Considering patients will require lifelong treatment with multiple doses of nusinersen per year, additional adverse effects due to chronic exposure to nusinersen are likely to emerge. In addition to nusinersen, several ASOs encompassing MOE modifications have been approved or in clinical development for the treatment of diverse pathological conditions [63, 64]. However, concerns still exist about adverse reactions and toxicity of ASO drugs [65]. Consistent with the previous findings [64], we observed less off-target effects of ASOs with MOE modifications than OMe modifications. Yet, our discovery of sequence-dependent MOE modification-specific off-target effects underscores the disadvantages of MOE modifications. Fortunately, at low concentrations, F18MOE/nusinersen is able to completely restore *SMN2* exon 7 splicing with minimal off-target effects. Based on our results, PMO emerges as a superior chemistry for splicing modulation if disadvantage of poor cell permeability is addressed. Our findings provide novel insights, including reduction of ASO size and incorporation of mixed modifications as potential strategies for developing future ASO-based drugs encompassing MOE modifications. While we tested mixed modifications carrying only MOE and OMe moieties, incorporation of other chemistries may produce a better alternative. Combination therapies involving low concentration of nusinersen and other small molecules could also serve as an alternative [51, 66–68]. While tolerance for mismatch base pairing by a therapeutic oligonucleotide is not desirable, it has unparalleled potential to reveal novel functional motifs related to the target sequence. Our discovery that diverse ISS-N1-like sequences potentially serve as ESEs underscores how the role of identical motifs changes, rather drastically, when presented in different contexts. Based on our findings, we envision a greater role of ASO-based strategies in uncovering unique splicing regulatory *cis-*elements, particularly large functional motifs that cannot be accurately predicted by available algorithms.

## Supplementary Material

ugag002_Supplemental_Files

## Data Availability

All major splice variants and sequences of key minigenes reported in this study can be accessed through NCBI; accession numbers are listed in Supplementary Table S2. RNA-Seq data can be accessed through NCBI GEO, accession number GSE307431.
