# Supplementary material for: A therapeutic antisense oligonucleotide encompassing 2′-O-methoxyethyl modification triggers unique perturbation of the transcriptome
Source: NAR Mol Med. 2026 Jan 6;3(1):ugag002. doi: 10.1093/narmme/ugag002 (PMC12805893; doi:10.1093/narmme/ugag002)

## SUPPLEMENTARY DATA

### Supplementary Figure legends

**Supplementary Figure S1. Specifications and quality control data for ASOs used in this study** (**A**) Specification sheet from IDT describing F18MOE. (**B**) Specification sheet from IDT describing ScrMOE. (**C**) Specification sheet from IDT describing F18OMe. (**D**) Specification sheet from IDT describing ScrOMe. (**E**) Mass spectrometry data from GeneTools verifying F20PMO purity. (**F**) Mass spectrometry data from GeneTools verifying ScrPMO purity. (**G**) Certificate of analysis from MedChemExpress verifying nusinersen quality and purity.

**Supplementary Figure S2. ISS-N1-targeting ASOs incorporating PMO modifications do not significantly alter expression of any off-target genes.** (**A**) Relative expression of genes predicted by RNA-Seq to be altered by F20PMO in a sequence-specific manner, as measured by qPCR. Identities of ASOs are indicated at the bottom (x axis). "C" indicates cells without any ASO treatment. Error bars represent standard error of the mean (SEM). (**B**) Relative expression of genes predicted by RNA-Seq to be altered by ScrPMO in a sequence-specific manner, as measured by qPCR. (**C**) Levels of transcripts predicted by RNA-Seq to be altered by both PMO-modified ASOs, as measured by qPCR.

**Supplementary Figure S3. Overrepresentation analysis of genes affected by ISS-N1-targeting ASOs.** (**A**) Top 10 Gene Ontology (GO) terms and Kyoto Encyclopedia of Genes and Genomes (KEGG) pathways that are enriched among genes upregulated upon treatment with F18OMe. The pathways are indicated at the left side of each graph and the level of statistical significance (-$\log_{10}$ transformed false discovery rate (FDR)) of each category is indicated on the x axis. (**B**) Top 10 GO terms, KEGG pathways, and chromosomal regions that are enriched among genes downregulated downregulated upon treatment with F18OMe. Grey bars indicate categories with FDR > 0.05. (**C**) Chromosomal regions that are enriched among genes that are upregulated upon treatment with F18MOE. (**D**) Top 10 GO terms that are enriched among genes that are downregulated upon treatment with F18MOE.

**Supplementary Figure S4. Comparison of the effects of F18MOE and commercially available nusinersen on differential gene expression.** (**A**) Relative expression of genes predicted by RNA-Seq to be downregulated by F18MOE in HeLa cells transfected with 100 nM ScrMOE, F18MOE, or nusinersen (Nus), as measured by qPCR. Identities of ASOs are indicated at the bottom (x axis). Abbreviations: C, cells without any ASO treatment; nus, nusinersen. Error bars represent standard error of the mean (SEM). * - $p < 0.05$, ** - $p < 0.01$. (**B**) Relative expression of genes predicted by RNA-Seq to be upregulated by F18MOE in HeLa cells transfected with 100 nM ScrMOE, F18MOE, or nusinersen, as measured by qPCR.

**Supplementary Figure S5. Validation of differential gene expression triggered by ISS-N1-targeting ASOs carrying MOE modifications in a sequence-independent manner.** (**A**) Relative expression of genes predicted by RNA-Seq to be downregulated by both F18MOE and ScrMOE, as measured by qPCR. Identities of ASOs are indicated at the bottom (x axis). Color coding for ASO concentrations is indicated at the top. "C" indicates cells without any ASO treatment. Error bars represent standard error of the mean (SEM). * - $p < 0.05$, ** - $p < 0.01$. (**B**) Relative expression of genes predicted by RNA-Seq to be upregulated by both F18MOE and ScrMOE, as measured by qPCR.

**Supplementary Figure S6. Validation of differential gene expression triggered by all ASOs containing PS backbones.** (**A**) Relative expression of genes predicted by RNA-Seq to be downregulated by F18MOE, ScrMOE, F18OMe, and ScrOMe, as measured by qPCR. Response to MOE-modified ASO treatments is depicted in the upper panels, whereas response to OMe-modified ASO treatments is depicted in the lower panels. Identities of ASOs are indicated at the bottom (x axis). Color coding for ASO concentrations is indicated at the top. "C" indicates cells without any ASO treatment. Error bars represent standard error of the mean (SEM). * - $p < 0.05$, ** - $p < 0.01$. (**B**) Relative expression of genes predicted by RNA-Seq to be upregulated by F18MOE, ScrMOE, F18OMe, and ScrOMe, as measured by qPCR.

**Supplementary Figure S7. Validation of differential gene expression triggered by F18MOE in multiple cell lines.** (**A**) Treatment of GM03813 cells with 100 nM ScrMOE or F18MOE by lipofectamine-mediated transfection. Left panel: Splicing pattern of *SMN2* exon 7 upon treatment

with 100 nM of the indicated ASOs. "C" indicates cells without any ASO treatment. "FL" indicates exon 7 inclusion, "ΔE7" indicates exon 7 skipping. Percentage skipping of exon 7 is indicated at the bottom of the gel. Experiment was performed in triplicate and all three replicates are shown. Middle panel: Relative expression of genes predicted by RNA-Seq to be downregulated by F18MOE as measured by qPCR. Color coding for ASO treatments are indicated at the top. * - $p < 0.05$, ** - $p < 0.01$. Right panel: Relative expression of genes predicted by RNA-Seq to be upregulated by F18MOE as measured by qPCR. (**B-D**) Treatment of HeLa (panel B), HEK293 (panel C), and SH-SY5Y cells (panel D) with 100 nM ScrMOE or F18MOE by lipofectamine transfection. Labeling, coloring, and layout are the same as in (A).

**Supplementary Figure S8. Location and local context of genes with altered expression/splicing upon F18MOE treatment.** A broad overview of all 24 chromosome types is shown. The locations of each upregulated, downregulated gene and genes harboring skipping and inclusion events that we performed validation for are given. For each gene, we also listed several flanking genes and examined our RNA-Seq data for upregulation or downregulation. Significant genes are color coded and labeled with symbols: +, upregulated; -, downregulated; &, exon inclusion, and *, exon skipping.

**Supplementary Figure S9. Splicing of exons predicted by RNA-Seq to undergo increased inclusion that were not strongly affected.** Genomic overview and splicing pattern of five exons predicted by RNA-seq to undergo increased inclusion after treatment by F18MOE. Exons are depicted as boxes, introns as lines/broken lines. Exon and intron sizes are indicated. Alternatively spliced exons are colored grey. Treatments are indicated at the top of the gel. "C" indicates cells without any ASO treatment. "FL" indicates exon inclusion, "ΔE_" indicates skipping of the indicated exon. Percentage skipping of relevant exons are indicated at the bottom of the gel.

**Supplementary Figure S10. Comparison of the splice-modulating effects of F18MOE and commercially available nusinersen.** Splicing pattern of *SMN1/2* exon 7 and seven off-target exons that undergo increased skipping upon treatment by F18MOE in HeLa cells transfected with increasing amounts of F18MOE or nusinersen. Treatments are indicated at the top of the

gel. “FL” indicates exon inclusion, “ΔE_” indicates skipping of the indicated exon. Percentage skipping of relevant exons are indicated at the bottom of the gel.

**Supplementary Figure S11. Potential annealing sites of F18MOE in off-target skipped exons.** The abridged sequences and flanking intron sequences of eight exons undergoing increased exon skipping upon treatment with F18MOE are given. Exon sequences are boxed and given in uppercase, intron sequences in lowercase. Numbering is indicated relative to the start of each exon. Splice sites and their respective strengths are indicated. Annealing position of F18MOE is shown, with canonical base pairs shown with black lines and wobble base pairs as open red circles.

**Supplementary Figure S12. Complete complementarity to the target sequence allows F20PMO to trigger efficient *POLR2H* exon 2 skipping.** Top panel: Annealing location of F20PMO in *POLR2H* exon 2 and mutated sequences designed to strengthen ASO annealing. Mutated bases are highlighted in green. Lower panels: Splicing pattern of transcripts generated from pPOLRH, endogenous *POLR2H*, and endogenous *SMN1/2* in cells conucleofected with pPOLR2H minigene and 6 µM of the indicated ASOs. Labeling is the same as in Supplementary Figure S7.

**Supplementary Figure S13. pPOLR2H studies performed in HeLa cells.** (**A**) Overview of pPOLR2H minigene. Exons, CMV promoter, and termination signal are depicted as boxes, introns as lines/broken lines. Sizes of exons and introns are indicated. (**B**) Splicing pattern of transcripts generated from pPOLR2H cotransfected with 100 nM of the indicated ASO. (**C**) Top panel: Overview of intron 1 deletions in pPOLR2H. Exons are shown as boxes, introns as solid black lines. Deleted sequences are indicated with dashed lines. Numbering is given relative to the closest exon. Lower panel: Splicing pattern of generated from pPOLR2H carrying intronic deletions cotransfected with 100 nM of the indicated ASO. (**D**) Left panel: Annealing location of F18MOE in *POLR2H* exon 2 and mutated sequences designed to weaken ASO annealing. Canonical base pairs are indicated with black lines. Wobble base pairs are indicated with open red circles. Mutated bases are highlighted in red. Right panel: Splicing pattern of transcripts generated by mutant pPOLR2H cotransfected with 100 nM of the indicated ASOs. (**E**) Left

panel: Annealing location of F18MOE in *POLR2H* exon 2 and mutated sequences designed to strengthen ASO annealing. Mutated bases are highlighted in green. Right panel: Splicing pattern of transcripts generated from mutant pPOLR2H cotransfected with increasing amounts of F18MOE (0, 5, 20, 100 nM). (**F**) Left panel: Annealing location of F18OMe in *POLR2H* exon 2 and mutated sequences designed to strengthen ASO annealing. Right panel: Splicing pattern of transcripts generated from mutant pPOLR2H cotransfected with increasing amounts of F18OMe (0, 5, 20, 100 nM).

**Supplementary Figure S14. Splicing pattern of additional hybrid minigenes and endogenous counterparts to hybrid minigenes.** (**A**) Splicing pattern of transcripts generated from hybrid minigenes cotransfected with 100 nM of the indicated ASO. Labeling is similar to Supplementary Figure S7. Abbreviations: Eh: hybrid exon, $Ih^{Up}$: upstream hybrid intron, $Ih^{Dn}$: downstream hybrid intron, Unsp – unspliced product, * - nonspecific PCR product, +I – intron retained products. (**B**) Splicing pattern of endogenous transcripts corresponding to exons used for hybrid minigenes. (**C**) Upper panels: Predicted annealing location of F18MOE in various target exons (WT) and mutated sequences (ISS) designed to strengthen ASO annealing. Coloring and labeling is the same as Supplementary Figure S9. Lower panels: Splicing pattern of transcripts generated from mutated hybrid minigenes cotransfected with 100 nM of the indicated ASO. (**D**) Splicing pattern of mutant minigenes generated in (C) in the presence of F18OMe. Coloring and labeling are the same as in (C).

**Supplementary Figure S15. Splicing pattern of endogenous transcripts expressed under conditions of hnRNPA1 and/or hnRNPA2B1 depletions.** (**A**) Splicing pattern of endogenous *SMN1* and *SMN2* exon 7 after depletion of hnRNPA1 and/or hnRNPA2B1. Transcripts derived from *SMN1* and *SMN2* are distinguished by digestion with the restriction endonuclease DdeI, which specifically cleaves *SMN2* but not *SMN2* due to a single nucleotide polymorphism in the recognition site. (**B**) Splicing pattern of endogenous *POLR2H* exon 2 after depletion of hnRNPA1 and/or hnRNPA2B1. (**C**) Splicing pattern of all other validated skipped exons after depletion of hnRNPA1 and/or hnRNPA2B1.

**Supplementary Figure S16. Short ASOs have similar effects in HeLa cells as HEK293 cells.** (**A**) Upper panel: Overview of F18MOE and shorter ASOs annealing to ISS-N1 within *SMN2* intron 7. ASOs are numbered, which is used throughout the figure. Lower panel: Splicing pattern of transcripts generated from pSMN2 in HEK293 cells cotransfected with the indicated ASOs. Treatments are indicated at the top of the gel. Labeling is the same as Figure 1C. (**B**) Upper panel: Overview of F18MOE and shorter ASO variants annealing to *POLR2H* exon 2 in the pPOLR2H minigene. Labeling and coloring is the same as in Figure 4C. Lower panel: Splicing pattern of transcripts generated from pPOLR2H minigene in HEK293 cells cotransfected with the indicated ASOs. Labeling is the same as in (A).

**Supplementary Figure S1A**

INTEGRATED DNA TECHNOLOGIES

**SPECIFICATION SHEET** WWW.IDTDNA.COM

**27-Sep-2021**

Order No. **17983262**

Ref. No. **313010541**

**Sequence -** F18N1 (F18OMe)

100 nmole DNA Oligo, 18 bases

**5'- mU*mC*mA* mC*mU*mU* mU*mC*mA* mU*mA*mA* mU*mG*mC* mU*mG*mG -3'**

**Properties**

*Tm (50mM NaCl)**: 40.5 °C
*GC Content:* 38.9%
*Molecular Weight:* 6,179.9
*nmoles/OD260:* 5.7
*ug/OD260:* 35.3
*Ext. Coefficient:* 175,200 L/(mole·cm)

**Amount Of Oligo**

| | | |
|---|---|---|
| 3.1 = | 18 | = 0.11 |
| $OD_{260}$ | nmoles | mg |

For 100 µM: add 180 µL

**Shipped To**

ERIC OTTESEN
IOWA STATE UNIVERSITY
2069A VET MED
AMES, IA 50011-1134
USA
5152941394
*Customer No.* 193575 PO No. PO-1104575

**Secondary Structure Calculations**

Lowest folding free energy (kcal/mole): -0.22 at 25 °C
Strongest Folding Tm: 28.7 °C
Secondary structure should not affect yield or purity for this oligo.

| **Oligo Base Types** | Quantity |
|---|---|
| 2' O-Methyl RNA bases | 18 |

| **Modifications and Services** | Quantity |
|---|---|
| HPLC Purification | 1 |
| Na+ Salt Exchange | 1 |
| Phosphorothioate Bond | 17 |

**Disclaimer**

See on reverse page notes (I) (II) & (III) for usage, label license, and product warranties

*Mfg. iD* 452192723

Labels - Peel here

313010541 IDT
E.OTTESEN
452192723 27-Sep-2021
F18N1
5'-mU*mC*mA* mC*mU*mU* mU*mC*mA*
mU*mA*mA* mU*mG*mC* mU*mG*mG-3'
MW= 6,179.9g/mol Tm= 40.5°C
3.1OD = 18nmol= 0.11mg

313010541 IDT
E.OTTESEN
452192723 27-Sep-2021
F18N1
5'-mU*mC*mA* mC*mU*mU* mU*mC*mA*
mU*mA*mA* mU*mG*mC* mU*mG*mG-3'
MW= 6,179.9g/mol Tm= 40.5°C
3.1OD = 18nmol= 0.11mg

**INSTRUCTIONS**

•Lyophilized contents may appear as either a translucent film or a white powder. This variance does not affect the quality of the oligo.

•Please centrifuge tubes prior to opening. Some of the product may have been dislodged during shipping.

*The Tm shown takes no account of $Mg^{2+}$ and dNTP concentrations. Use the OligoAnalyzer® Program at www.idtdna.com/scitools to calculate accurate Tm for your reaction conditions.

M

## Supplementary Figure S1B

IDT
INTEGRATED DNA TECHNOLOGIES

**SPECIFICATION SHEET** WWW.IDTDNA.COM

**18-Oct-2021**

Order No. **18036353**

Ref. No. **314971127**

**Sequence** - 18OmeSCR (ScrOMe) — 100 nmole DNA Oligo, 18 bases

**5'- mC*mG*mA* mC*mG*mC* mU*mA*mC* mU*mU*mA* mU*mU*mA* mU*mU*mG -3'**

### Properties

*Tm (50mM NaCl)**: 35.7 °C
*GC Content*: 38.9%
*Molecular Weight*: 6,179.9
nmoles/OD260: 5.6
*ug/OD260*: 34.8
*Ext. Coefficient*: 177,700 L/(mole·cm)

### Amount Of Oligo

| | | |
|---|---|---|
| 5.9 = | 33.2 | = 0.21 |
| $OD_{260}$ | nmoles | mg |

For 100 µM: add 332 µL

### Shipped To

ERIC OTTESEN
IOWA STATE UNIVERSITY
2069A VET MED
AMES, IA 50011-1134
USA
5152941394
*Customer No. 193575* *PO No. PO-1107690*

### Secondary Structure Calculations

Lowest folding free energy (kcal/mole): 1.01 at 25 °C
Strongest Folding Tm: 0.4 °C
Secondary structure should not affect yield or purity for this oligo.

| Oligo Base Types | Quantity |
|---|---|
| 2' O-Methyl RNA bases | 18 |

| Modifications and Services | Quantity |
|---|---|
| HPLC Purification | 1 |
| Na+ Salt Exchange | 1 |
| Phosphorothioate Bond | 17 |

### Disclaimer

See on reverse page notes (I) (II) & (III) for usage, label license, and product warranties

*Mfg. ID454906617*

Labels - Peel here

314971127 IDT
E.OTTESEN
454906617 18-Oct-2021
18OmeSCR
5'- mC*mG*mA* mC*mG*mC* mU*mA*mC* mU*mU*mA* mU*mU*mA* mU*mU*mG -3'
MW= 6,179.9g/mol Tm= 35.7 °C
5.9OD = 33.2nmol= 0.21mg

314971127 IDT
E.OTTESEN
454906617 18-Oct-2021
18OmeSCR
5'- mC*mG*mA* mC*mG*mC* mU*mA*mC* mU*mU*mA* mU*mU*mA* mU*mU*mG -3'
MW= 6,179.9g/mol Tm= 35.7 °C
5.9OD = 33.2nmol= 0.21mg

### I N S T R U C T I O N S

•Lyophilized contents may appear as either a translucent film or a white powder. This variance does not affect the quality of the oligo.

•Please centrifuge tubes prior to opening. Some of the product may have been dislodged during shipping.

*The Tm shown takes no account of $Mg^{2+}$ and dNTP concentrations. Use the OligoAnalyzer® Program at www.idtdna.com/scitools to calculate accurate Tm for your reaction conditions.

M

# Supplementary Figure S1C

INTEGRATED DNA TECHNOLOGIES

OLIGONUCLEOTIDE SPECIFICATION SHEET

27-Jul-2021

Order No. **17833151**

Ref. No. **307123613**

**Sequence -** A10-27_MOE (A18MOE) — 100 nmole DNA Oligo, 0 bases

**5'- /52MOErT/*/i2MOErC/*/i2MOErA/* /i2MOErC/*/i2MOErT/*/i2MOErT/* /i2MOErT/*/i2MOErC/*/i2MOErA/ * /i2MOErT/*/i2MOErA/*/i2MOErA/* /i2MOErT/*/i2MOErG/*/i2MOErC/* /i2MOErT/*/i2MOErG/*/32MOErG/ -3'**

**Properties**

*Tm (50mM NaCl)**: 0.0 °C
*GC Content:* 38.9%
*Molecular Weight:* 7,127.2
*nmoles/OD260:* 5.7
*ug/OD260:* 40.7
*Ext. Coefficient:* 175,200 L/(mole·cm)

**Amount Of Oligo**

| | | |
|---|---|---|
| 4.6= | 26.3 | =0.19 |
| $OD_{260}$ | nmoles | mg |

For 100 µM: add 263 µL

**Shipped To**

ERIC OTTESEN
IOWA STATE UNIVERSITY
2069A VET MED
AMES, IA 50011-1134
USA
5152941394
*Customer No.* 193575 *PO No.* PO-1095339

| **Oligo Base Types** | Quantity |
|---|---|

| **Modifications and Services** | Quantity |
|---|---|
| HPLC Purification | 1 |
| Na+ Salt Exchange | 1 |
| 3' 2-MethoxyEthoxy G | 1 |
| 5' 2-MethoxyEthoxy T | 1 |
| Int 2-MethoxyEthoxy A | 4 |
| Int 2-MethoxyEthoxy G | 2 |
| Int 2-MethoxyEthoxy MeC | 4 |
| Int 2-MethoxyEthoxy T | 6 |
| Phosphorothioate Bond | 17 |

**Disclaimer**

*Mfg. ID* 440772619

307123613 IDT
E.OTTESEN
440772619 27-Jul-2021
A10-27_MOE
[illegible]
MW= 7,127.2g/mol Tm= 0.0°C
4.6OD = 26.3nmol = 0.19mg

307123613 IDT
E.OTTESEN
440772619 27-Jul-2021
A10-27_MOE
[illegible]
MW= 7,127.2g/mol Tm= 0.0°C
4.6OD = 26.3nmol = 0.19mg

**I N S T R U C T I O N S**

•Lyophilized contents may appear as either a translucent film or a white powder. This variance does not affect the quality of the oligo.

•Please centrifuge tubes prior to opening. Some of the product may have been dislodged during shipping.

*The Tm shown takes no account of $Mg^{2+}$ and dNTP concentrations. Use the OligoAnalyzer® Program at www.idtdna.com/scitools to calculate accurate Tm for your reaction conditions.

M

**Supplementary Figure S1D**

IDT®
INTEGRATED DNA TECHNOLOGIES
OLIGONUCLEOTIDE SPECIFICATION SHEET

27-Jul-2021

Order No. **17833151**

Ref. No. **307123614**

**Sequence -** SCRM18_MOE (ScrMOE) 100 nmole DNA Oligo, 0 bases

**5'- /52MOErC/*/i2MOErG/*/i2MOErA/* /i2MOErC/*/i2MOErG/*/i2MOErC/* /i2MOErT/*/i2MOErA/*/i2MOErC/* /i2MOErT/*/i2MOErT/*/i2MOErA/* /i2MOErT/*/i2MOErT/*/i2MOErA/* /i2MOErT/*/i2MOErT/*/32MOErG/ -3'**

**Properties**

*Tm (50mM NaCl)*:* 0.0 °C
*GC Content:* 38.9%
*Molecular Weight:* 7,127.2
*nmoles/OD260:* 5.6
*ug/OD260:* 40.1
*Ext. Coefficient:* 177,700 L/(mole·cm)

**Amount Of Oligo**

7.9 = 44.3 = 0.32
$OD_{260}$ nmoles mg

For 100 µM: add 443 µL

**Shipped To**

ERIC OTTESEN
IOWA STATE UNIVERSITY
2069A VET MED
AMES, IA 50011-1134
USA
5152941394
*Customer No.* 193575 PO No. PO-1095339

| **Oligo Base Types** | Quantity |
|---|---|

| **Modifications and Services** | Quantity |
|---|---|
| HPLC Purification | 1 |
| Na+ Salt Exchange | 1 |
| 3' 2-MethoxyEthoxy G | 1 |
| 5' 2-MethoxyEthoxy MeC | 1 |
| Int 2-MethoxyEthoxy A | 4 |
| Int 2-MethoxyEthoxy G | 2 |
| Int 2-MethoxyEthoxy MeC | 3 |
| Int 2-MethoxyEthoxy T | 7 |
| Phosphorothioate Bond | 17 |

**Disclaimer**

*Mfg. ID* 440751619

307123614 IDT
E.OTTESEN
440751619 27-Jul-2021
SCRM18_MOE
[illegible]
MW= 7,127.2g/mol Tm= 0.0°C
7.9OD = 44.3nmol= 0.32mg

307123614 IDT
E.OTTESEN
440751619 27-Jul-2021
SCRM18_MOE
[illegible]
MW= 7,127.2g/mol Tm= 0.0°C
7.9OD = 44.3nmol= 0.32mg

**I N S T R U C T I O N S**

•Lyophilized contents may appear as either a translucent film or a white powder. This variance does not affect the quality of the oligo.

•Please centrifuge tubes prior to opening. Some of the product may have been dislodged during shipping.

*The Tm shown takes no account of $Mg^{2+}$ and dNTP concentrations. Use the OligoAnalyzer® Program at www.idtdna.com/scitools to calculate accurate Tm for your reaction conditions.

M

## Supplementary Figure S1E

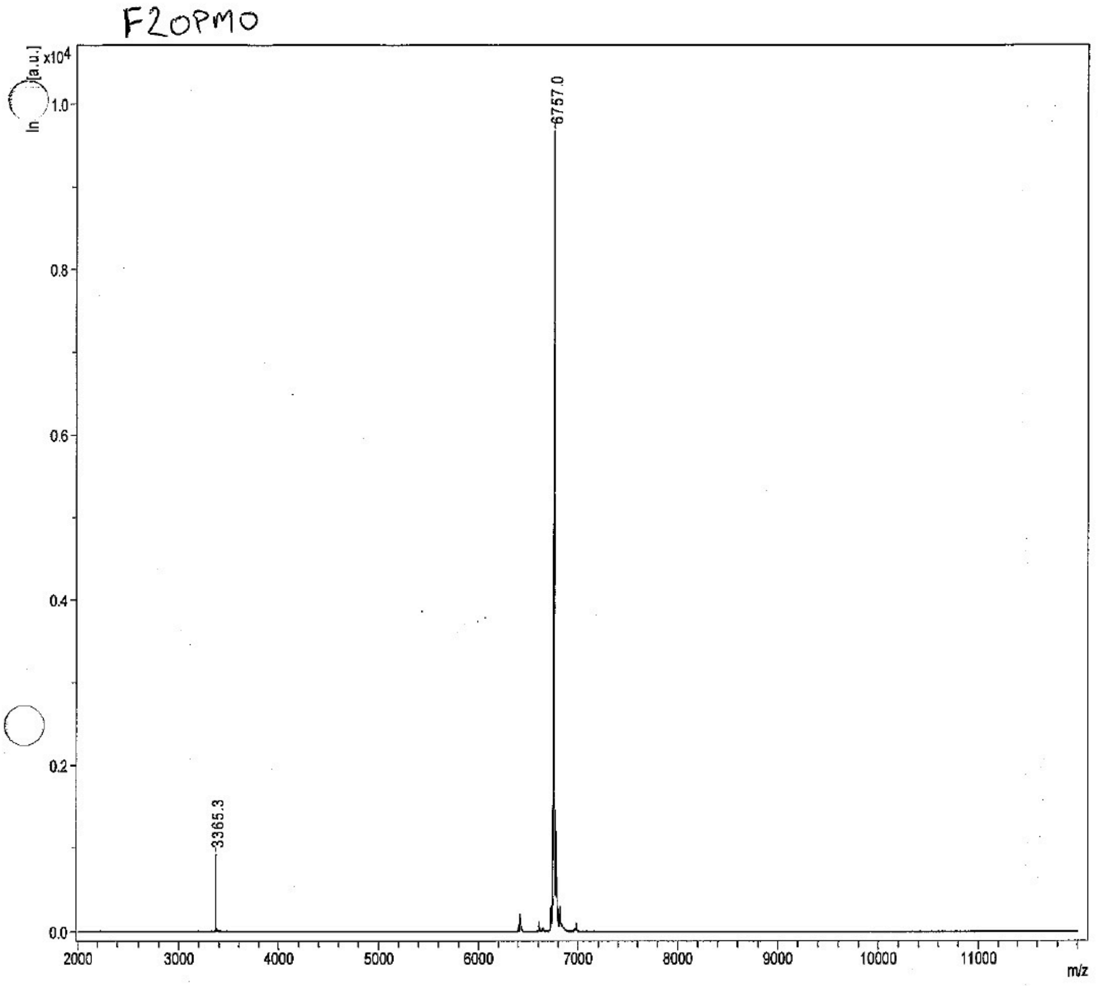


D:\Customer Mass Spec\2021\September\29Sep21A\26-29Sep21A\0_C2\1\1SLin

2021-10-01T11:22:39.388-07:00

**Supplementary Figure S1F**

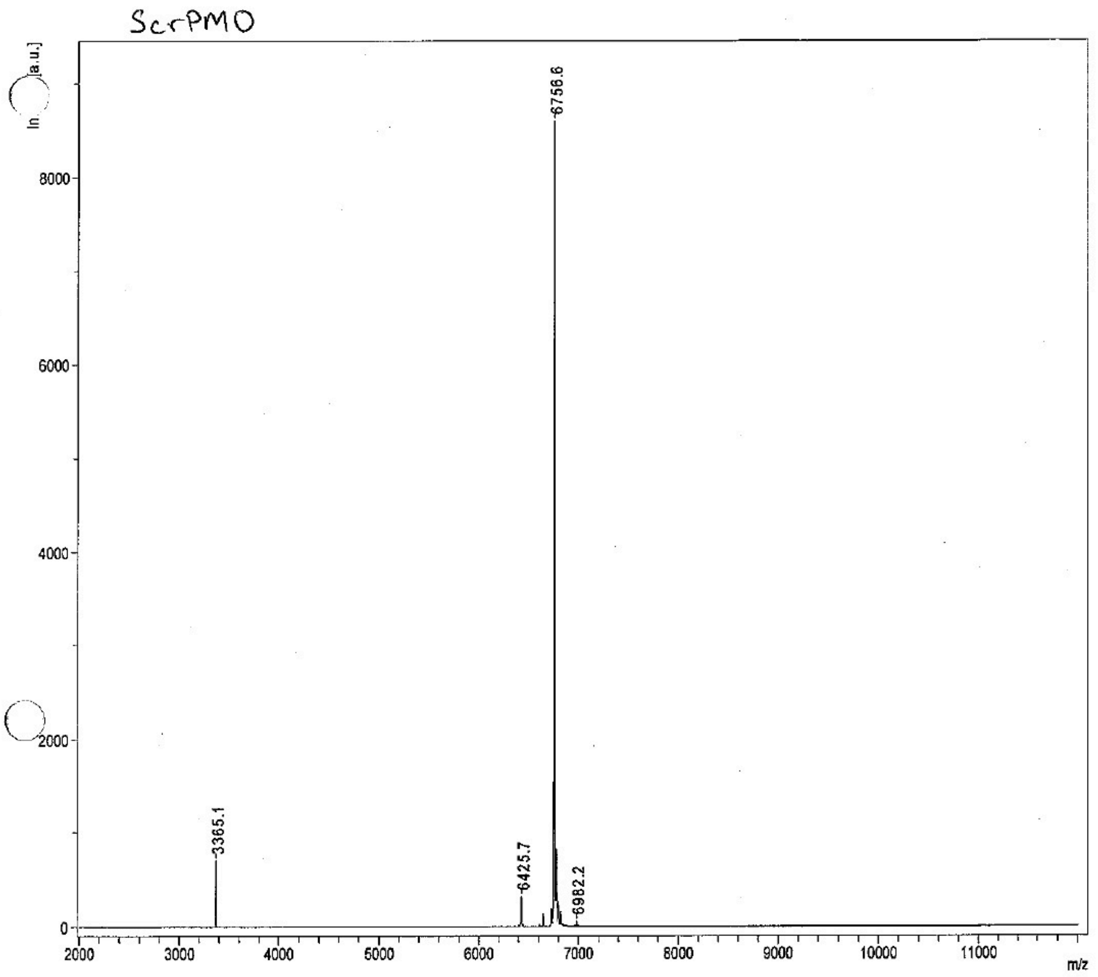


D:\Customer Mass Spec\2021\September\29Sep21A\27-29Sep21A\0_C3\1\1SLin

2021-10-01T11:22:43.675-07:00

**Supplementary Figure S1G**

MCE®
MedChemExpress

# Certificate of Analysis

Inhibitors • Screening Libraries • Proteins

## Nusinersen

| | |
|---|---|
| **Cat. No.:** | HY-112980 |
| **CAS No.:** | 1258984-36-9 |
| **Batch No.:** | 781004 |
| **Chemical Name:** | RNA, [2'-O-(2-methoxyethyl)](P-thio)(m5U-m5C-A-m5C-m5U-m5U-m5U-m5C-A-m5U-A-A-m5U-G -m5C-m5U-G-G) |

### PHYSICAL AND CHEMICAL PROPERTIES

| | |
|---|---|
| **Molecular Formula:** | $C_{234}H_{340}N_{61}O_{128}P_{17}S_{17}$ |
| **Molecular Weight:** | 7127.30 |
| **Storage:** | -20°C, sealed storage, away from moisture<br>* In solvent : -80°C, 6 months; -20°C, 1 month (sealed storage, away from moisture) |

**Chemical Structure:**

Nusinersen

### ANALYTICAL DATA

| | |
|---|---|
| **Appearance:** | White to off-white (Solid) |
| **LCMS:** | Consistent with structure |
| **Purity (LCMS):** | 97.79% |
| **Conclusion:** | The product has been tested and complies with the given specifications. |

**Caution: Product has not been fully validated for medical applications. For research use only.**

Tel: 609-228-6898 Fax: 609-228-5909 E-mail: tech@MedChemExpress.com
Address: 1 Deer Park Dr, Suite F, Monmouth Junction, NJ 08852, USA

**Supplementary Figure S2**

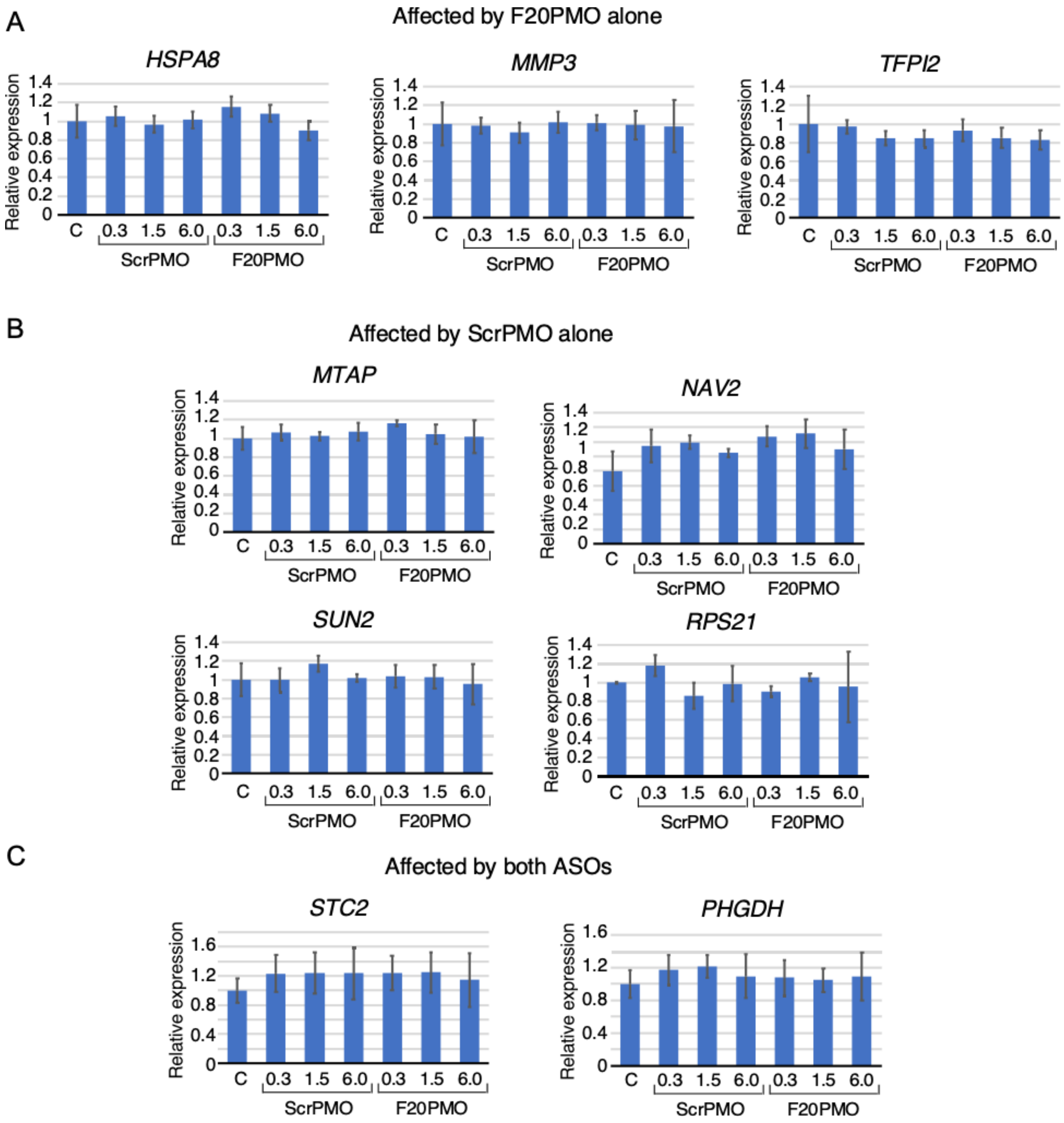

**Supplementary Figure S3**

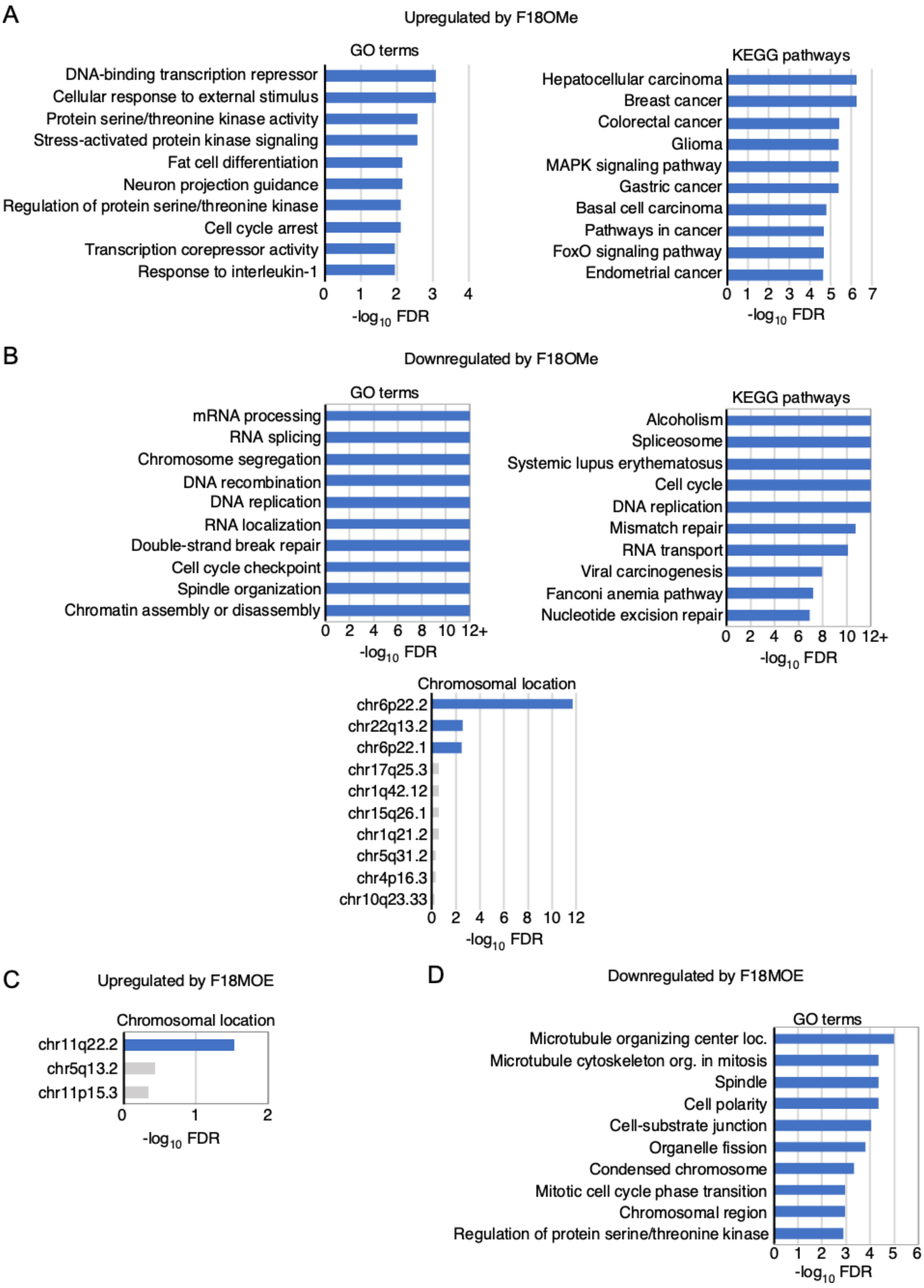

## Supplementary Figure S4

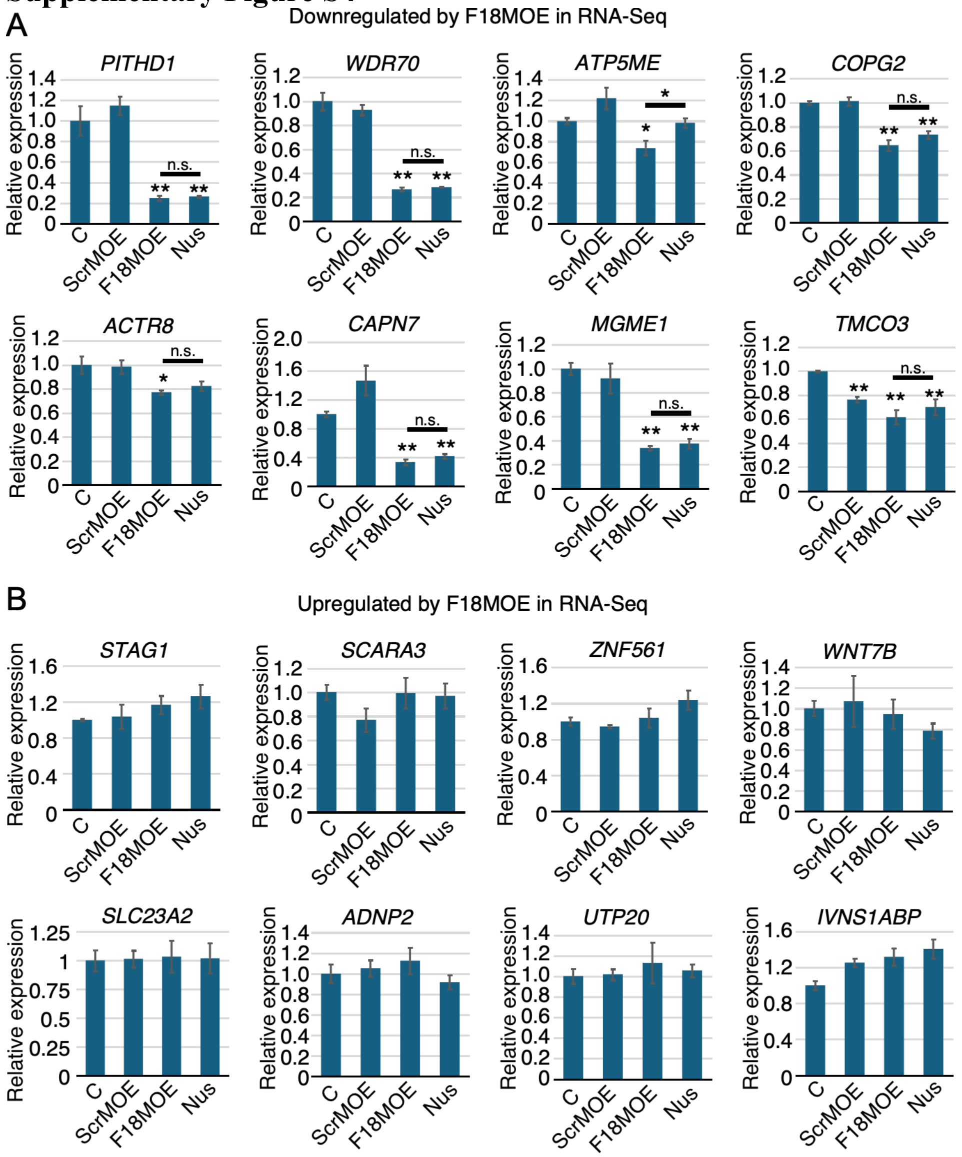

**Supplementary Figure S5**

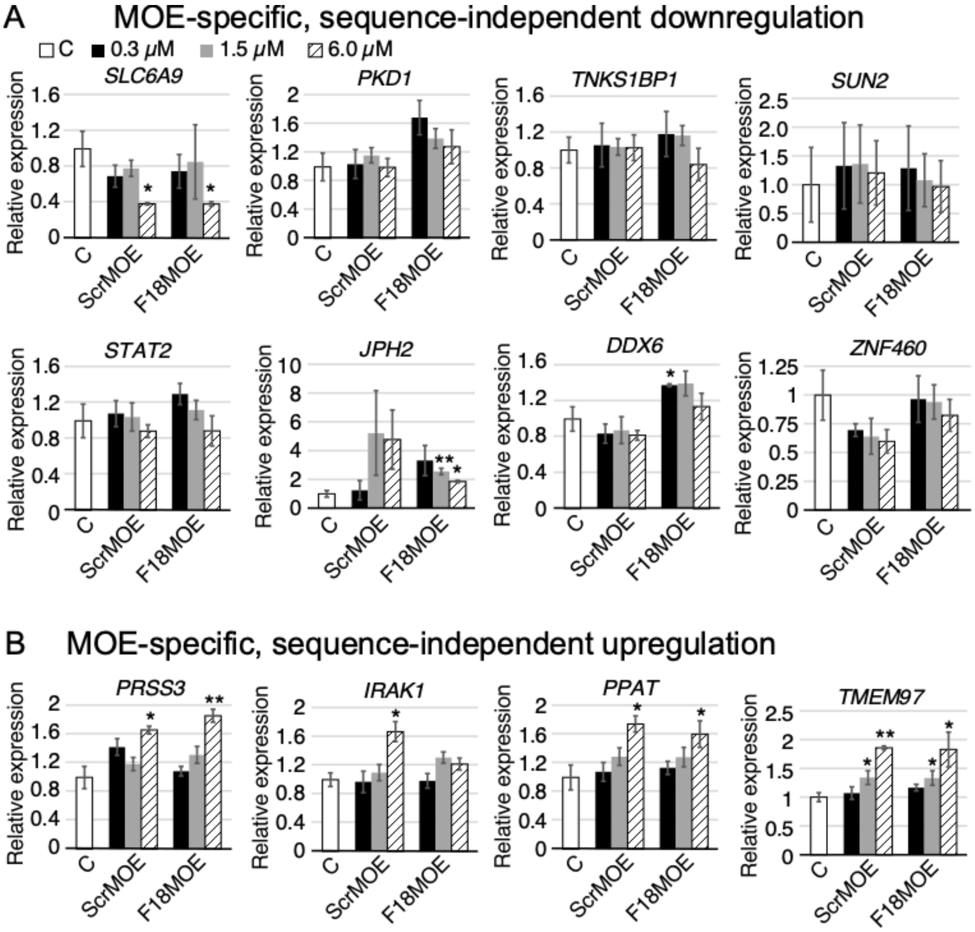

**Supplementary Figure S6**

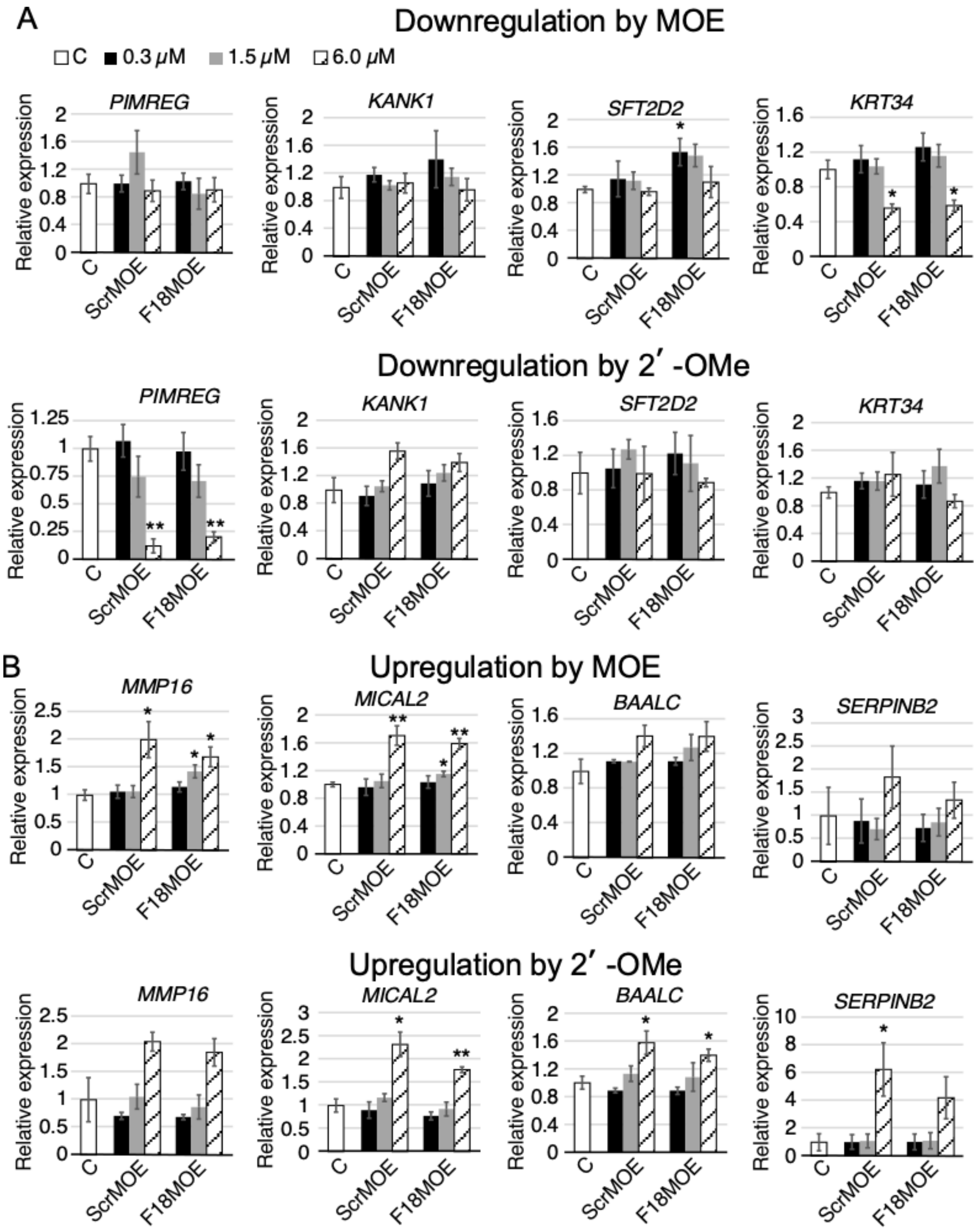

**Supplementary Figure S7**

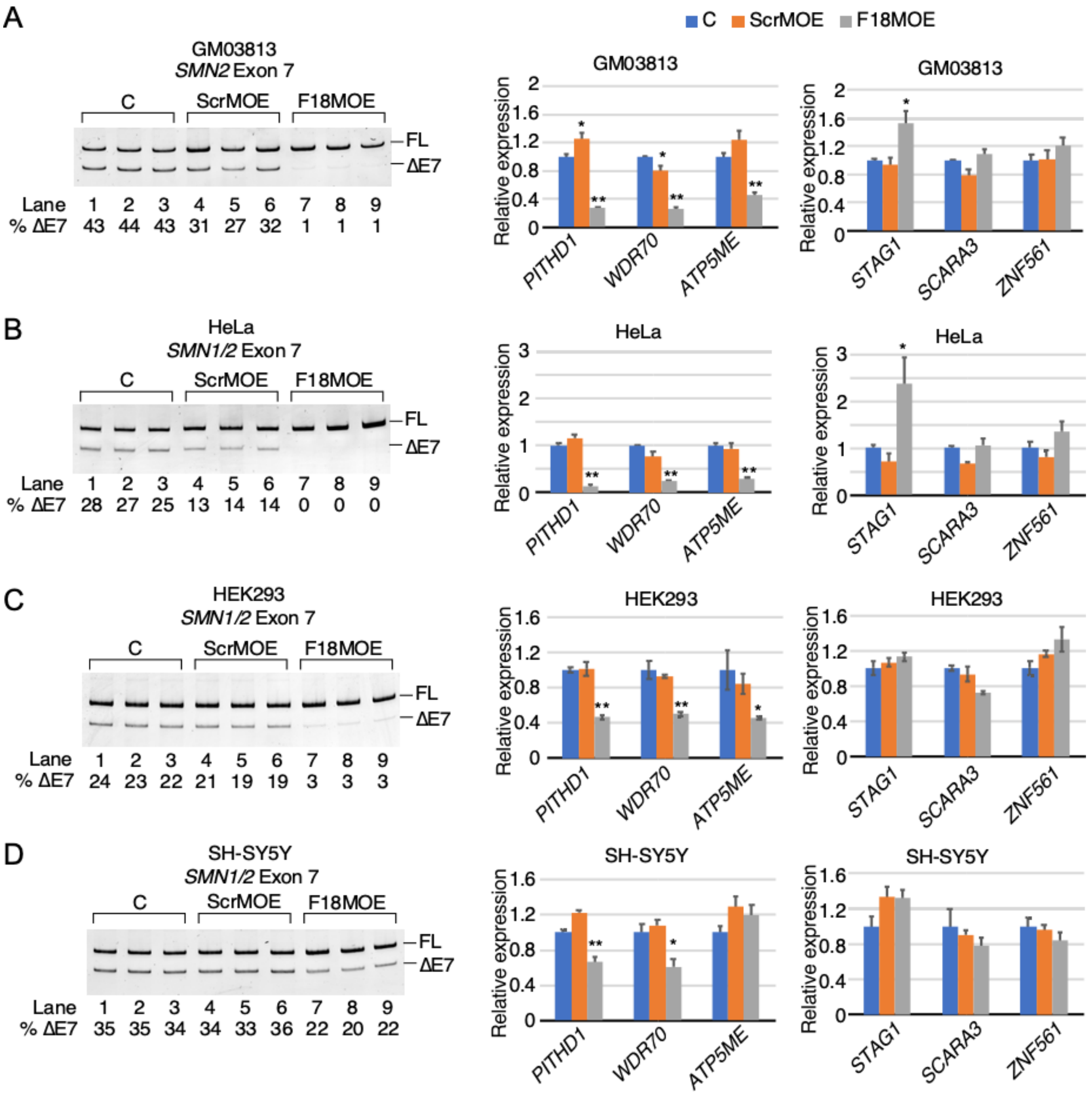

## Supplementary Figure S8

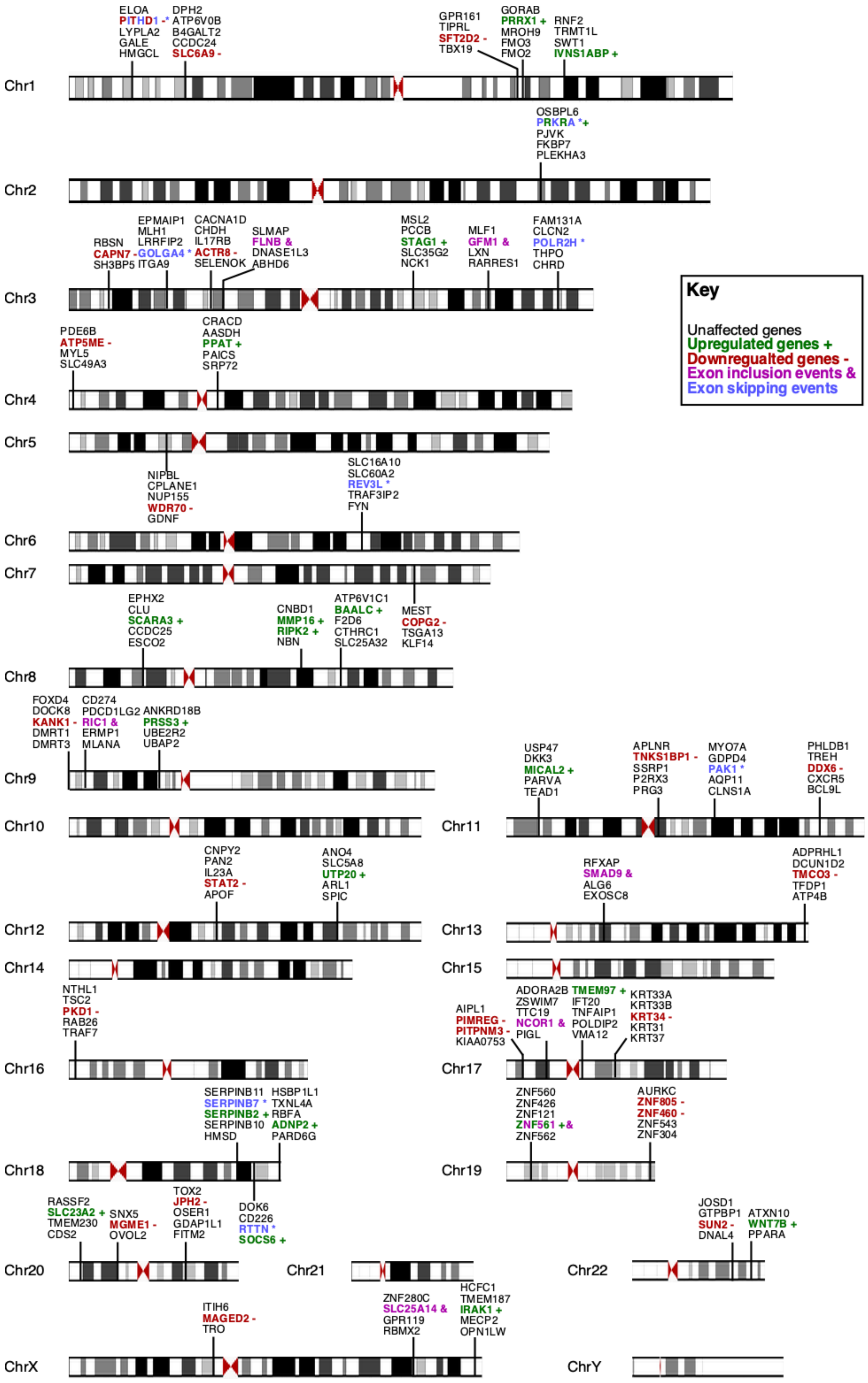

**Supplementary Figure S9**

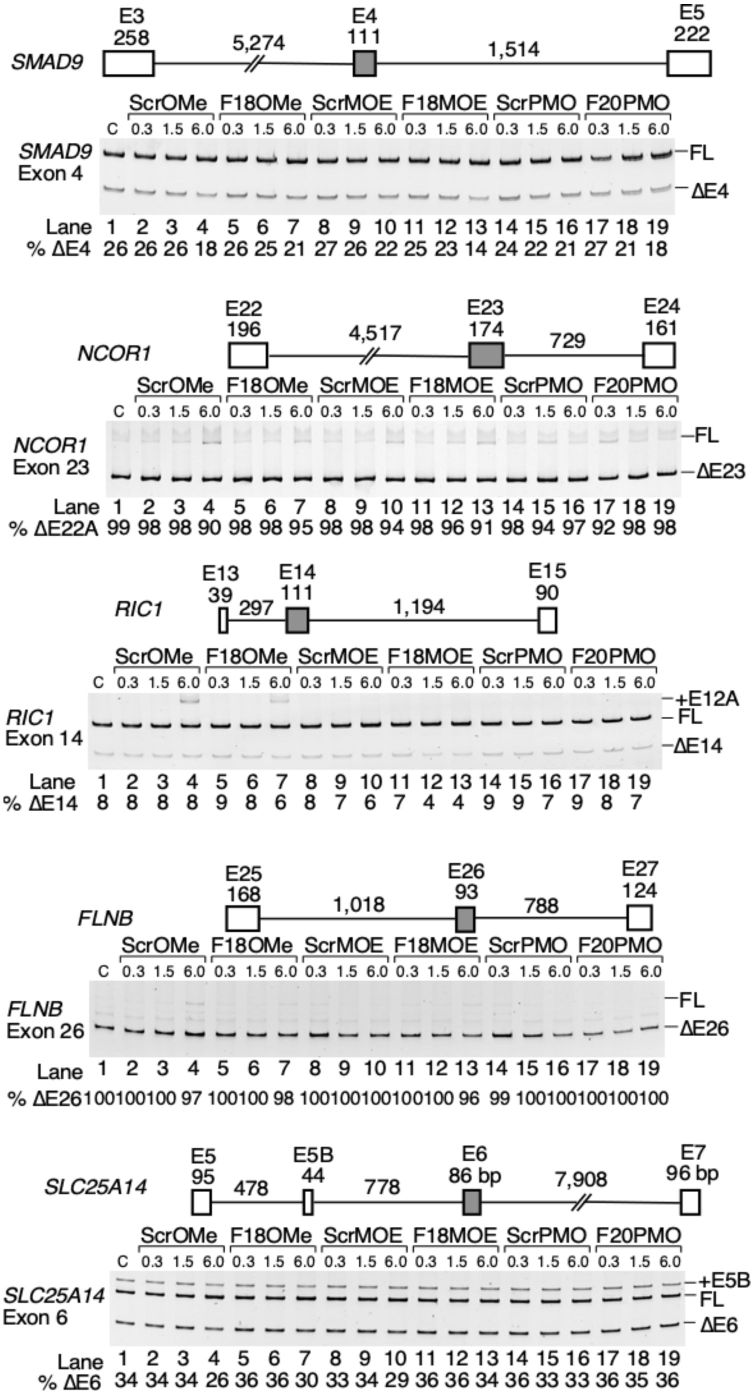

**Supplementary Figure S10**

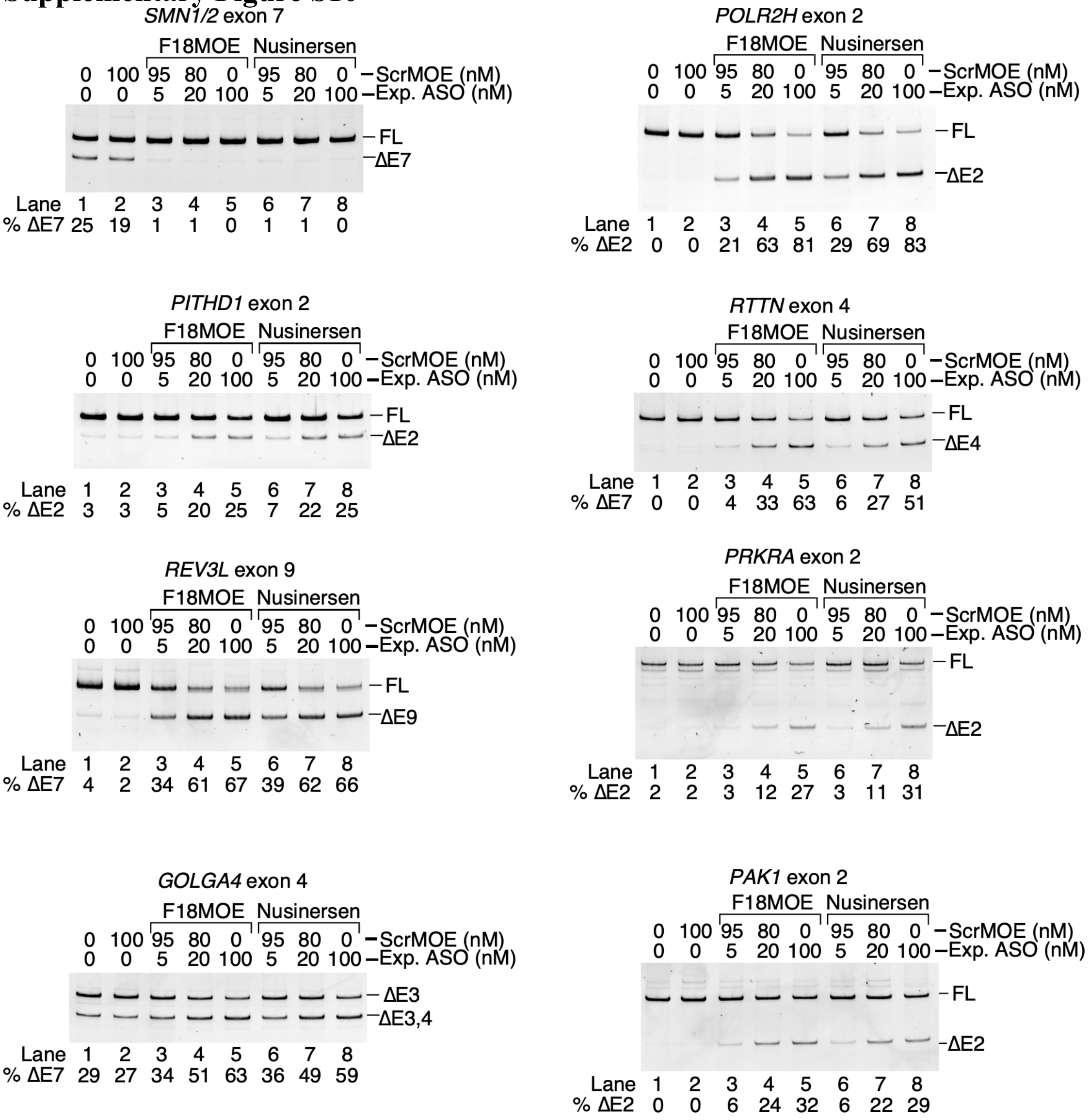

## Supplementary Figure S11

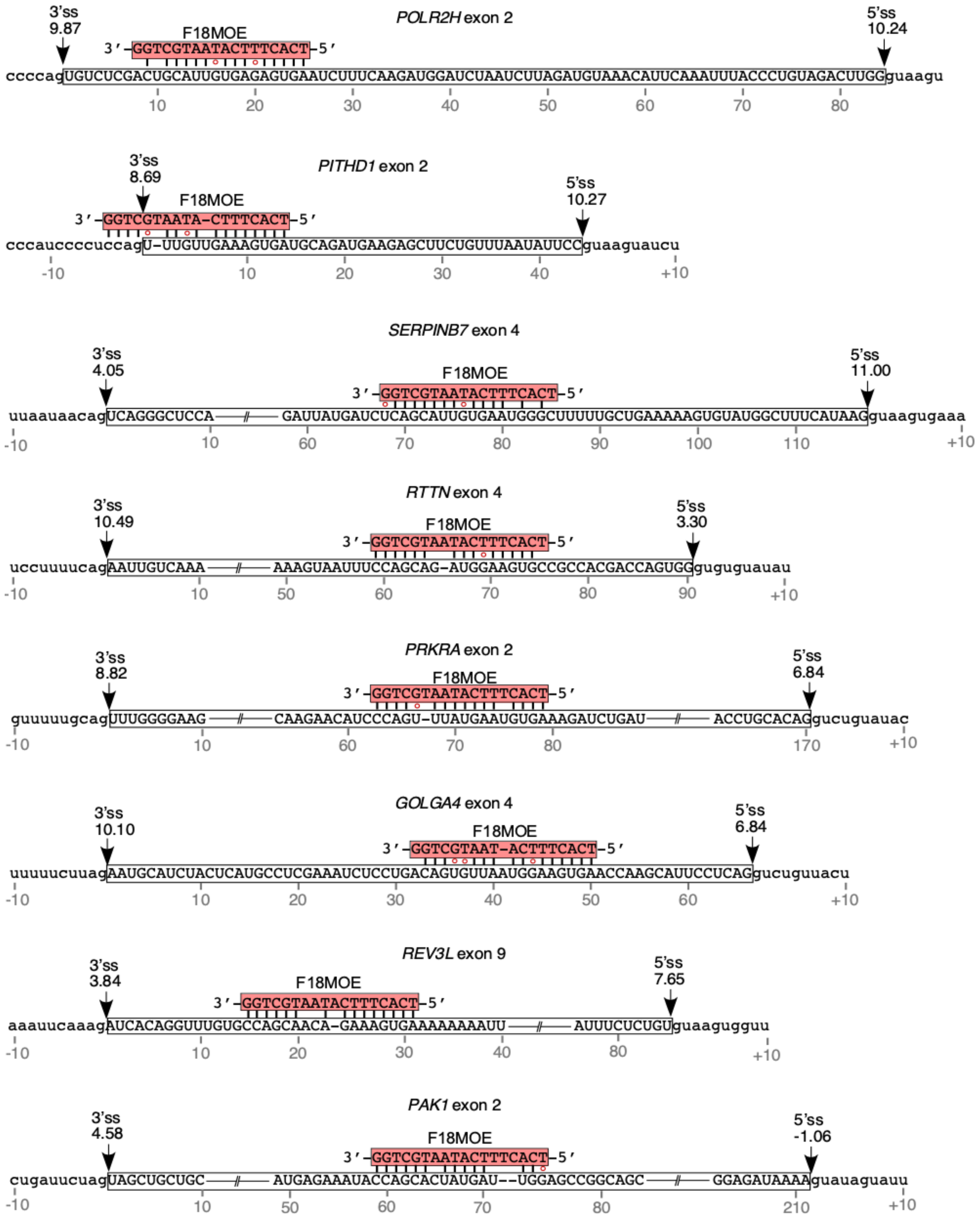

## Supplementary Figure S12

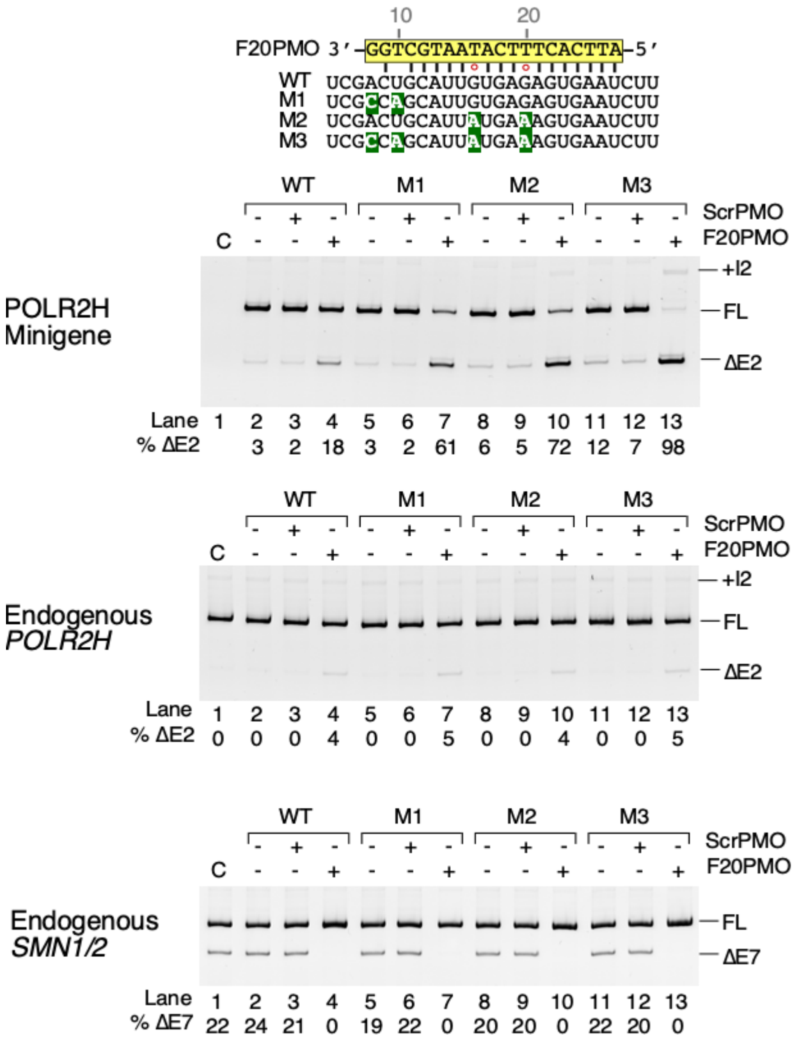

**Supplementary Figure S13**

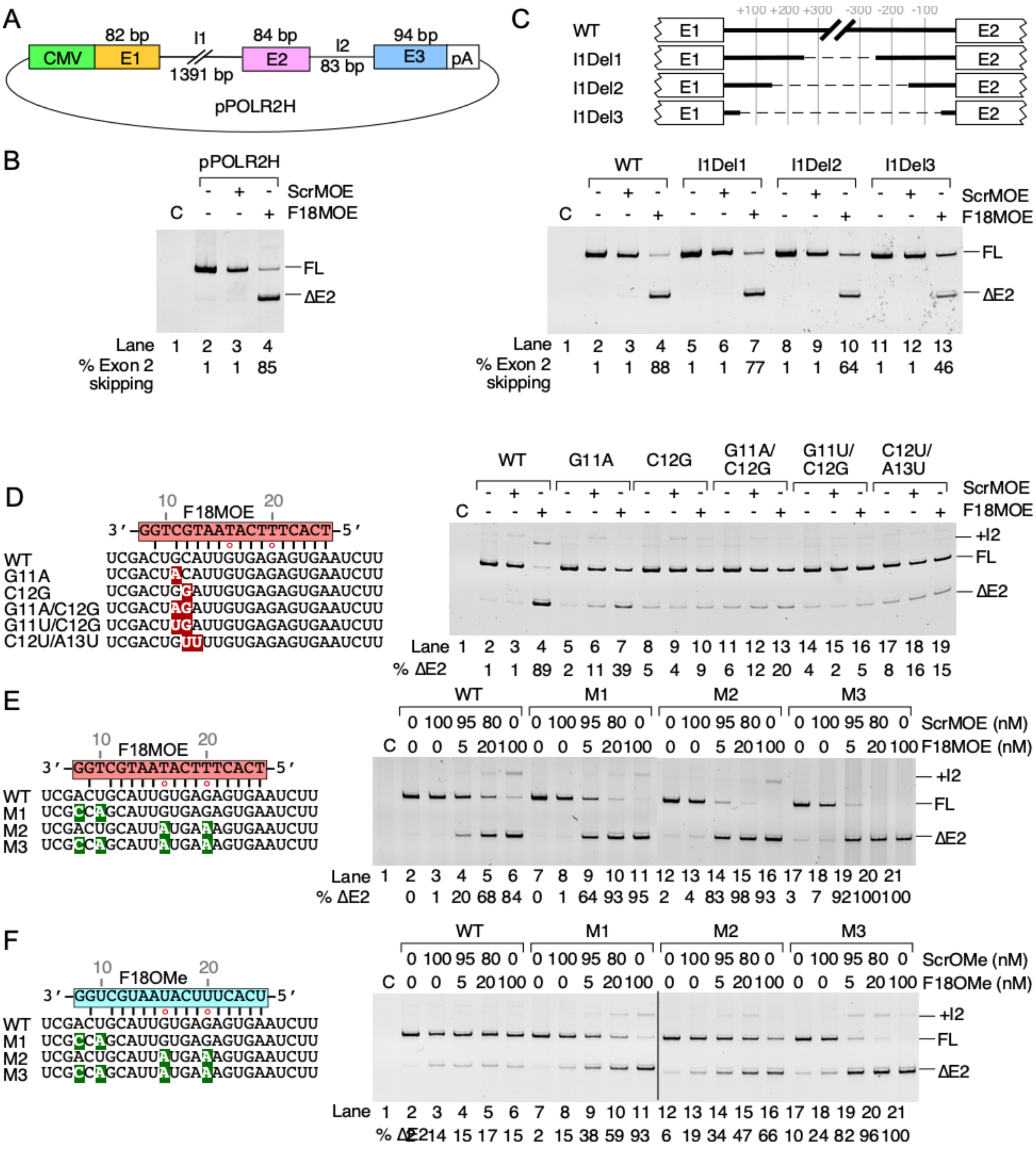

## Supplementary Figure S14

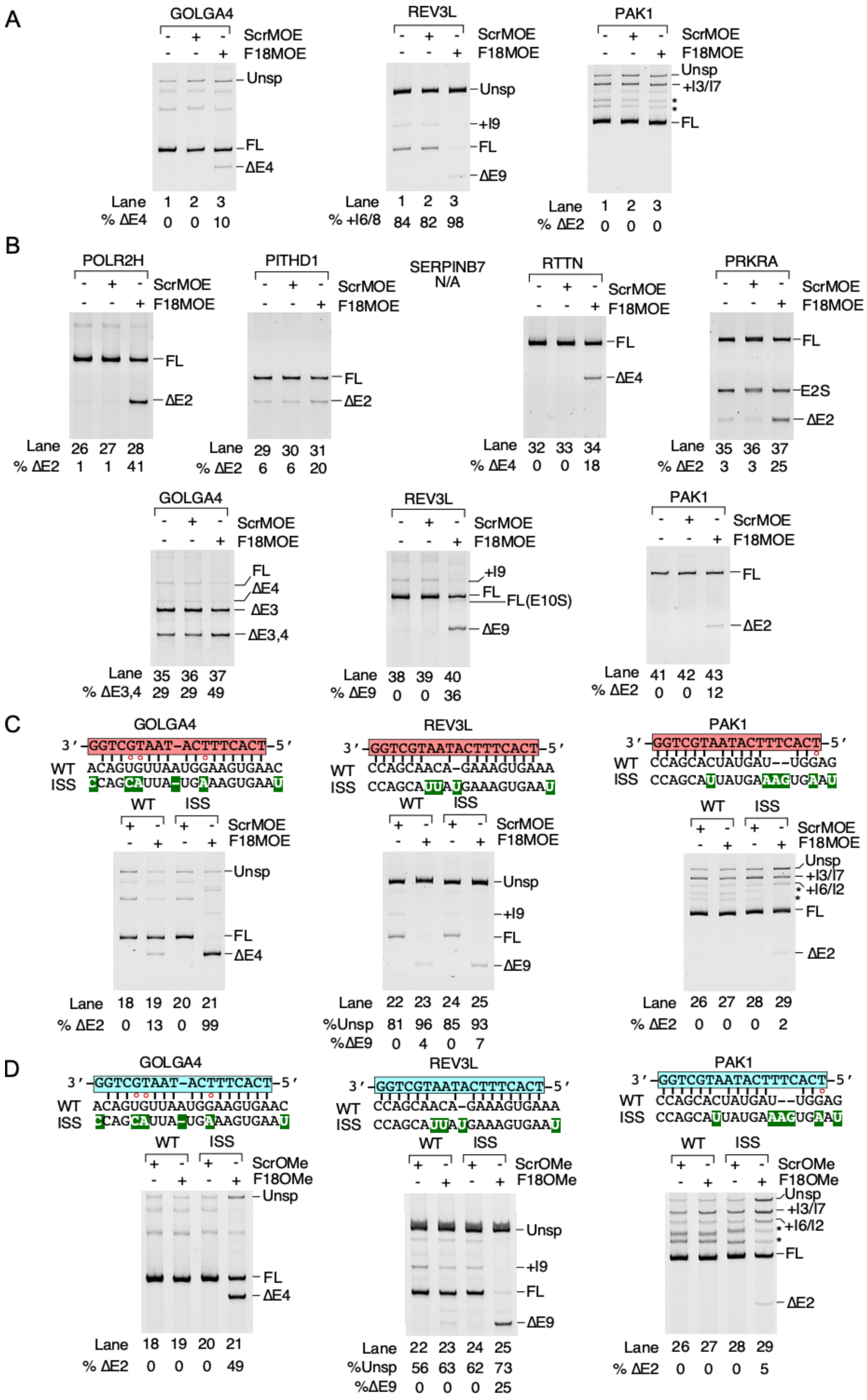

# Supplementary Figure S15

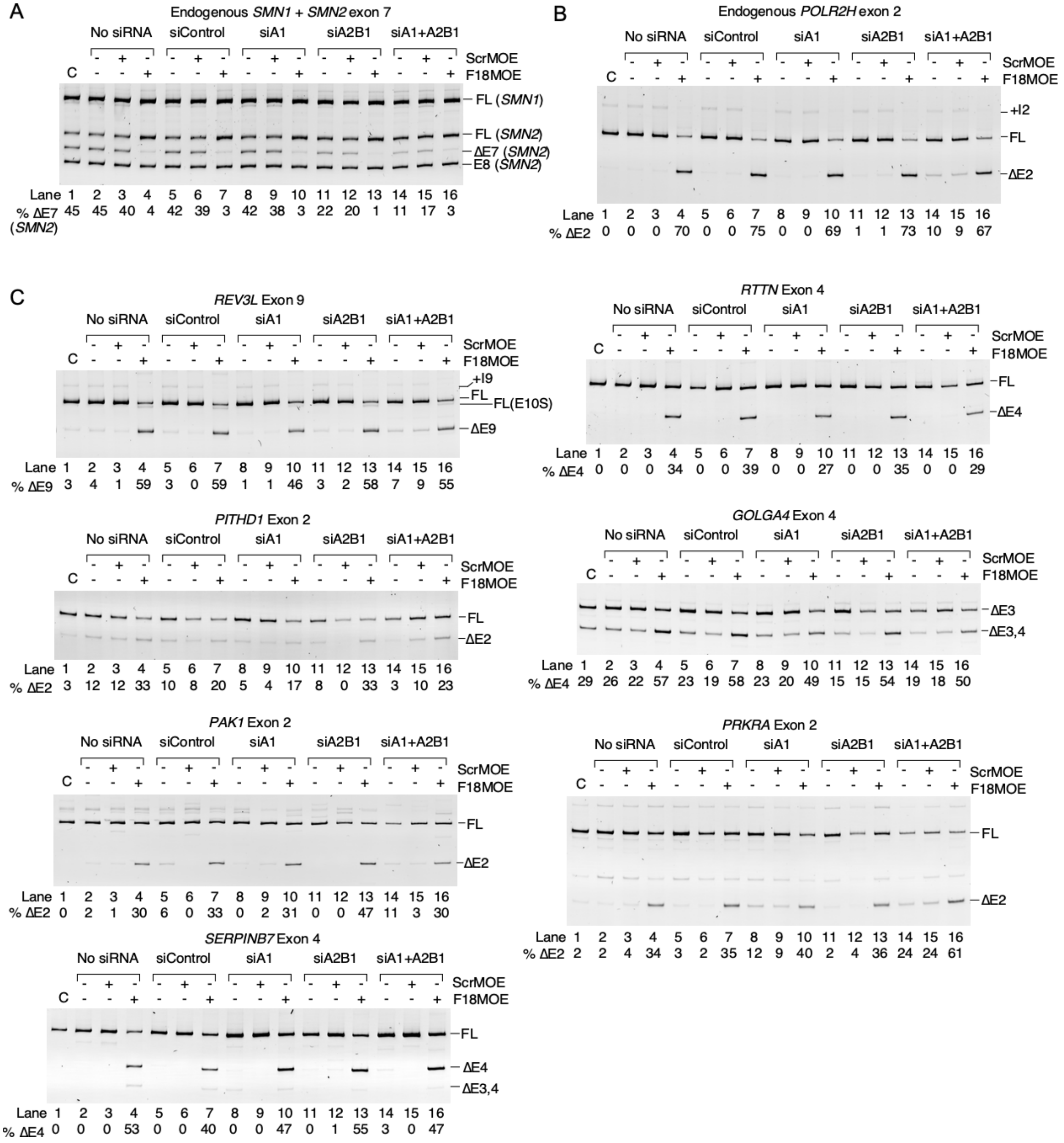

## Supplementary Figure S16

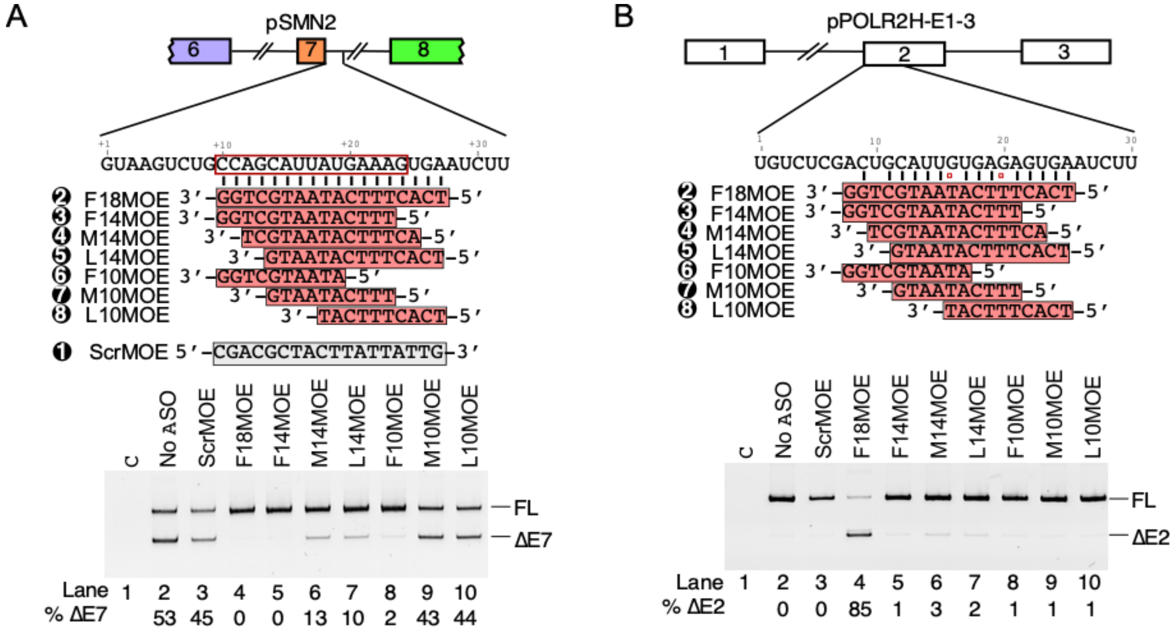

Supplement: ugag002_Supplemental_Files [file ugag002_supplemental_files.zip › Supplementary Data 12-18-2025.pdf]
